# Functional analysis of thiamine pyrophosphate-responsive riboswitches in human bacterial pathogens of the ESKAPE group using a dual-luciferase reporter gene assay

**DOI:** 10.1128/jb.00308-25

**Published:** 2025-10-09

**Authors:** Anna Hübenthal, Vipul Panchal, Ruth Brenk, Matthias Mack

**Affiliations:** 1 Department of Biotechnology, Institute for Technical Microbiology, Hochschule Mannheim 120168, Mannheim, Germany; 2 Department of Biomedicine, University of Bergen 1658, Bergen, Norway; University of Illinois Chicago, Chicago, Illinois, USA

**Keywords:** riboswitches, ESKAPE pathogens, thiamine

## Abstract

Thiamine pyrophosphate (TPP)-responsive riboswitches are genetic elements in bacteria that regulate the expression of genes coding for proteins involved in the biosynthesis and transport of thiamine (vitamin B_1_). Following uptake, cytoplasmic thiamine is converted to TPP, which serves as a cofactor for enzymes of central metabolic pathways such as glycolysis, the tricarboxylic acid cycle, and the pentose phosphate pathway, and it is the level of TPP (and not thiamine) that is sensed by TPP riboswitches. TPP riboswitches are the most widespread riboswitches in bacteria. Their key roles in metabolism combined with their absence in humans make them potential targets for antibiotics, whereby the focus of the present study was pathogenic bacteria of the ESKAPE group: *Enterococcus faecium*, *Staphylococcus aureus*, *Klebsiella pneumoniae*, *Acinetobacter baumannii*, *Pseudomonas aeruginosa,* and *Enterobacter* spp. As a first step toward the development of novel TPP riboswitch-targeting antimicrobials to treat infections caused by ESKAPE organisms, we characterized various TPP riboswitches present in these bacteria. We developed a dual-luciferase reporter gene assay to monitor riboswitch activity and found that most of the predicted TPP riboswitches indeed were functional regulators and responded to TPP. In contrast to the *Escherichia coli thiC* TPP riboswitch, TPP riboswitches from ESKAPE bacteria were found not to respond to the synthetic thiamine analog pyrithiamine. One TPP riboswitch of *K. pneumoniae* was examined in detail with regard to the effect of pyrithiamine. Site-directed mutagenesis experiments identified specific nucleotides responsible for the non-response to pyrithiamine, and this should be useful in developing novel TPP riboswitch-targeting antimicrobials.

**IMPORTANCE:**

Riboswitches are RNA molecules that control important processes in bacteria. Infections with pathogens of the ESKAPE group are common, and we are trying to find new ways to fight these bacteria. Small molecules can be designed to bind to riboswitches and optimally block their activity. In the present work, we have analyzed the thiamine pyrophosphate (TPP) riboswitches of ESKAPE pathogens with respect to small molecule binding. For this purpose, we developed a dual-luciferase reporter gene assay. Most of the predicted TPP riboswitches were indeed functional regulators and are thus targets for new anti-infectives. The small molecule pyrithiamine does not block all TPP riboswitches tested, and we found a structural basis for this behavior.

## INTRODUCTION

**Fig 1:**
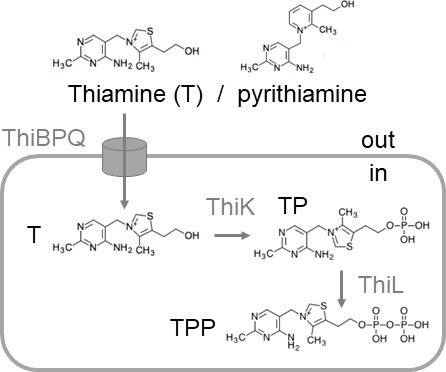
Probable uptake and metabolism of thiamine and the synthetic structural analog pyrithiamine. Following uptake by the ABC transporter ThiBPQ, thiamine is accepted as a substrate by thiamine kinase ThiK (EC 2.7.1.89) and thiamine-monophosphate kinase ThiL (EC 2.7.4.16), which in concert lead to pyrophosphorylation (16). The scheme only shows the formation of thiamine-monophosphate (TP) and thiamine pyrophosphate (TPP). It is very likely that pyrithiamine is metabolized in a similar way (16). TPP is an enzyme cofactor, whereas pyrithiamine pyrophosphate (structure not shown) is a potentially less active or inactive cofactor analog (16). The names of the gene products are identical for *Escherichia coli* and *Klebsiella pneumoniae* (20).

Metabolite-sensing bacterial riboswitches are RNAs typically located in the 5′-untranslated parts of mRNAs and control the synthesis of metabolism-related proteins. Riboswitches consist of an aptamer domain and a downstream expression platform. While the aptamer is responsible for binding a specific small molecule ligand, the expression platform translates the ligand-induced conformational change into a response of the transcription or translation machinery (1). Various types of expression platforms are exploited by riboswitches, but the two most common mechanisms involve regulation of premature transcription termination (“transcription control”) and control of ribosome binding to mRNAs (“translation control”) (2). To date, 55 different classes of riboswitches have been discovered that selectively sense small molecules or elementary ions, and it is believed that there are many more (3). Across these known 55 classes and based on the type of ligand, riboswitches can be divided into several groups, the biggest of which comprises riboswitches that bind and respond to the enzyme cofactors flavin mononucleotide (FMN), adenosylcobalamin, or TPP, metabolites derived from RNA nucleotides or their precursors. TPP riboswitches appear to be the most widespread riboswitches and control the expression of genes involved in thiamine biosynthesis and uptake (3, 4). Once present in the cytoplasm, thiamine is enzymatically converted to TPP, which serves as an enzyme cofactor (Fig. 1). The TPP riboswitches controlling the expression of *Escherichia coli thiC* (encoding phosphomethylpyrimidine synthase, EC 4.1.99.17) and *thiM* (encoding hydroxyethylthiazole kinase, EC 2.7.1.50) were the first TPP riboswitches to be experimentally validated (5). They have been subjected to in-depth studies, including structural analysis and examination of ligand recognition (6–10), and thus appeared to represent ideal models for our present study. The *thiBPQ* genes encode ABC transporters for thiamine and TPP in Enterobacteriaceae (11), and their expression is also controlled by riboswitches. The structures of known TPP riboswitch aptamers are highly conserved, with the RNA forming two binding pockets (7). One binding pocket formed by the P2 and P3 stem of the riboswitch RNA encloses the pyrimidine moiety of TPP (12), while the negatively charged pyrophosphate group of TPP is coordinated by two Mg^2+^ ions and forms direct and indirect (water-mediated) bonds with nucleotides of the P4 and P5 stem (13, 14). It appears that the central thiazole ring of TPP plays a minor role in ligand recognition, as its replacement by other positively charged heterocyclic rings did not have a significant effect on binding affinity (10). This is in line with the observation that the synthetic TPP analog pyrithiamine pyrophosphate (PTPP) (Fig. 1) (15), in which a pyrimidine ring replaces the central thiazole ring of thiamine, binds to specific aptamer portions of the TPP riboswitch controlling *Bacillus subtilis tenA* (encoding thiaminase II, EC 3.5.99.2) with high affinity (16). Due to their wide distribution in the genomes of pathogens, their high sequence conservation, and their importance for the regulation of important metabolic pathways, the TPP riboswitches were considered promising targets for a new class of anti-infectives (4, 17, 18). This inspired us to further investigate these genetic elements. The focus of the present study was pathogenic bacteria of the ESKAPE group: *Enterococcus faecium*, *Staphylococcus aureus*, *Klebsiella pneumoniae*, *Acinetobacter baumannii*, *Pseudomonas aeruginosa,* and *Enterobacter* spp. (19). We employed a dual-luciferase reporter gene assay to experimentally validate and characterize putative TPP riboswitches from ESKAPE pathogens as potential drug targets. We found that most of these TPP riboswitches, unlike the *E. coli* TPP riboswitches, did not respond to pyrithiamine, i.e., pyrithiamine pyrophosphate. In the case of the *thiC* riboswitch of *K. pneumoniae,* we could show that a single nucleotide in the P3 stem region of this RNA was responsible for this effect.

## MATERIALS AND METHODS

### Cultivation of bacterial strains

*E. coli* DH5α (thiamine auxotrophic) and *E. coli* MG1655 (thiamine prototrophic) were grown in M9 minimal medium supplemented with 0.1% (wt/vol) casamino acids and 0.4% (wt/vol) glucose. When not otherwise indicated, thiamine-HCl (20 nM) was added to cultures of *E. coli* DH5α, as this strain is thiamine auxotrophic. Ampicillin (100 µg/mL) was added to recombinant *E. coli* strains containing reporter plasmids. Cultures of *K. pneumoniae* (1 mL) were grown in a 24-well plate in M9 minimal medium supplemented with 0.1% (wt/vol) casamino acids and 0.4% (wt/vol) glucose. Plates were placed in a humidity chamber and incubated in a plate reader (Tecan Spark, Männedorf, Switzerland) at 37°C with double-orbital shaking at an amplitude of 6. Optical density at 600 nm was measured automatically every 45 minutes.

### Plasmid and strain construction

All oligonucleotides used for plasmid and strain construction, as well as for sequencing, can be found in Table S1. The dual-luciferase plasmid was constructed using pPrib-RibDG-RFN-luc as a backbone (21), which in turn is based on the high-copy number (colE1 origin of replication) single-luciferase reporter plasmid pT7luc (Promega, Mannheim, Germany). In this plasmid, firefly luciferase *lucF* served as a reporter gene. A codon-adapted (to *E. coli*), second luciferase gene from *Renilla reniformis* (*lucR*) was introduced to pPrib-RibDG-RFN-luc downstream of *lucF* to create a dual-reporter plasmid. The gene *lucR* was placed under the control of the strong constitutive promoter pOXB15 (derived from the *recA* promoter by random mutagenesis, Oxford Genetics Limited, Oxford, UK), and *lucF* expression was normalized to *lucR* expression. The *rrnB* T1 terminator sequence was amplified by PCR from chromosomal DNA of *E. coli* using primer pairs adding *Sac*I and *Xho*I sites. The resulting PCR product was ligated to *Sac*I and *Xho*I-restricted pPrib-RibDG-RFN-luc to generate pPrib-RFN-luc-t. A *KpnI* restriction site was added to pPrib-RFN-luc-t 12 base pairs downstream of the new *Xho*I site by amplifying the whole plasmid using the overlapping primers 5′-ATA GGT CGG TAC CGA GCT CCC AAA AAA AAA A-3′ and 5′-GGT ACC GAC CTA TCA GAA CTC GAG TAC TCA G-3′ and ligation of the resulting PCR product. Codon-adapted *lucR* in combination with pOXB15 was obtained as one custom DNA string (Thermo Fisher Scientific, Waltham, MA, USA). A PCR product was generated using a modifying primer pair and the DNA string as a template, which allowed ligation to *Xho*I/*Kpn*I-restricted pPrib-luc.t, creating pPrib-RFN-Dluc. The *Hin*dIII site upstream of *lucR* in pPrib-RFN-Dluc was replaced by *Nco*I using site-directed mutagenesis. To remove the FMN riboswitch sequence from pPrib-RibDG-RFN-luc (21), complementary oligonucleotides containing a ribosomal binding site and the start codon ATG were used as follows. The two oligonucleotides (10 µM each) were mixed in 1× NEBuffer 2 (New England Biolabs GmbH, Frankfurt a. M., Germany) and annealed by heating the mixture to 98°C for 5 minutes before slowly cooling it down to room temperature. The obtained linker was ligated to *Nco*I/*Bam*HI-restricted pPrib-RFN-Dluc, creating the plasmid pPrib-Dluc. Another pair of complementary oligonucleotides was annealed to create a second linker with a blunt end and a sticky end to be ligated to *Pvu*II/*Nco*I-restricted pPrib-Dluc to remove the *rib* promoter sequence and finally create the translational fusion plasmid pDluc. A stop codon, a ribosomal binding site upstream of *lucF,* and a start codon for *lucF* were introduced to pDluc by site-directed mutagenesis to generate the transcriptional fusion plasmid pDlucTC. The *thiC* promoter region of *E. coli* was amplified and ligated to *Pvu*II/*Nco*I-restricted pDluc and pDlucTC, respectively, creating plasmids pDluc::pEc01 and pDlucTC::pEc01. The TPP riboswitch regions from different bacterial species, including the promoters, riboswitches, and the ribosomal binding sites (RBSs) in combination with the first five codons of the downstream genes, were obtained as custom DNA constructs cloned into pMA (Thermo Fisher Scientific, Waltham, MA, USA). The riboswitches Ab01, Kp04, Eb01, Eb03, Ms01, Ms02, Pa01, and Sa02 were amplified from these templates using modifying primer pairs. The resulting DNA fragments were coupled to *Nco*I/*Bam*HI-restricted pDluc::pEc01 and (except for Sa02) pDlucTC::pEc01. The TPP riboswitches Ec01, Ab01, Ef02, Kp04, Ms01, Kp01, Kp10, and Kp11 were amplified from custom DNA templates in combination with their native promoters. These DNA fragments were ligated to *Pvu*II/*Bam*HI-treated pDluc and pDlucTC. Promoter control constructs were also obtained as custom DNA constructs and amplified using the same primer pairs. Promoter control constructs lacked riboswitch sequences and consisted only of the native riboswitch promoter and the RBS, including the first few codons of the downstream genes (Table S2). The reporter plasmids for the mutated Kp04 riboswitch versions were generated from pDluc::Kp04 by site-directed mutagenesis. Site-directed mutagenesis was also used to insert the P3 stem sequence of Kp04 into pDluc::Ec01. All cloning steps and newly constructed plasmids were checked and validated by DNA sequencing. Plasmids were propagated using *E. coli* DH5α and used to transform either *E. coli* DH5α or *E. coli* MG1655. For the construction of the β-galactosidase-based reporter gene plasmid pHA191, pUC19 was used as a backbone. In the first step, the *lac* promoter was deleted from this plasmid using site-directed mutagenesis and overlapping primers, generating pUC19del. The *lacZ* reporter gene was amplified by PCR from pDG268 and ligated to *Xba*I/*Eco*RI-treated pUC19del, creating pHA191. The TPP riboswitch regions Ec01 from *E. coli* and Kp04 from *K. pneumoniae,* including the promoters, ribosomal binding sites, and the first few codons of the downstream genes, were amplified from genomic DNA and inserted between the *Hin*dIII and *Xba*I restriction sites to give pHA191. The riboswitch regions and the control inserts were obtained as custom DNA constructs ligated to the GeneArt pMA plasmid (Thermo Fisher Scientific, Waltham, MA, USA).

### Plasmid site-directed mutagenesis

Plasmid mutagenesis was performed using the QuikChange Lightning Site-Directed Mutagenesis Kit (Agilent, Santa Clara, CA, USA), following the manufacturer’s instructions. The Agilent QuikChange Primer Design tool was used to design the complementary oligonucleotides for the integration of the respective insertions, deletions, or nucleotide changes.

### Dual-luciferase assay

Overnight cultures (1 mL) of *E. coli* DH5α or MG1655 containing pDluc or pDlucTC to test the different TPP riboswitches were grown in M9 minimal medium in 24-well plates (VWR International GmbH, Darmstadt, Germany) at 37°C and 160 rpm. The next day, these overnight cultures were used to inoculate 2 mL test cultures (triplicate) to an initial OD_600_ of 0.1 in a 12-well plate (VWR International GmbH, Darmstadt, Germany). These test cultures were supplemented with 10 µM of either thiamine or pyrithiamine and incubated at 37°C and 180 rpm for 4 hours. Cells were harvested by centrifugation, and cell pellets were washed once with 500 µL of 1× phosphate-buffered saline (PBS). Following another round of centrifugation, cell pellets were suspended in 100–200 µL of 1× passive lysis buffer (PLB, Dual Luciferase Reporter Assay System, Promega, Mannheim, Germany), and cell suspensions were kept for 15 minutes on ice. The total protein content was then determined according to Bradford. Lysed cells were diluted with 1× PLB to a total protein level of 20 µg/mL. The diluted samples (20 µL) were transferred to white flat-bottom 96-well microplates (Greiner Bio-One GmbH, Frickenhausen, Germany). These plates were incubated in a Tecan Spark reader for 10 minutes at room temperature before starting luminescence measurement (LucF and LucR). Meanwhile, the connected injectors were flushed three times with H_2_O. Subsequently, injector A was primed with Luciferase Assay II Reagent (LAR II; Dual Luciferase Reporter Assay System, Promega) and injector B with Stop & Glo reagent (Dual Luciferase Reporter Assay System, Promega). For measurement, 100 µL LAR II was injected followed by a 3-second waiting period, before the luminescence measurement was performed with automatic attenuation, an integration time of 10,000 ms and a settling time of 0 ms. Subsequently, 100 µL of Stop & Glo reagent was injected per well, and luminescence was measured using the same parameters as before. The output was recorded in counts per second.

### β**-**galactosidase assay for testing thiamine analogs other than pyrithiamine

Pre-cultures of the test strains containing pHA191 were grown in 1 mL of M9. The cultures were incubated overnight in a 24-well plate at 37°C and shaking at 160 rpm. The OD_600_ of each overnight culture was determined and transferred to a 1.5 mL microcentrifuge tube. The cells were collected by centrifugation at 6,000 × *g* for 5 minutes and washed once with M9. The cell pellets were resuspended in M9, and the suspensions were used to inoculate 200 µL of fresh M9 in a 96-well plate to an initial OD_600_ of 0.1. Thiamine and different thiamine analogs dissolved in DMSO were added to the cultures (1 mM), and the plates were incubated for 4 hours at 37°C with shaking at 180 rpm. Following incubation, 100 µL of the cultures was transferred to a fresh 96-well plate, and the OD_600_ was determined. Ten microliters of a 1:1 mixture of PopCulture reagent (Merck KGaA, Darmstadt, Germany) and a lysozyme solution (50 mg in 1 mL Z buffer) (22) were added to each well. The plate was incubated at room temperature for 15 minutes to promote cell lysis. Following cell lysis, 15 µL of each cell lysate was mixed with 155 µL Z buffer containing 0.135% of dithiothreitol. A volume of 30 µL of ortho-nitrophenyl-β-galactoside (ONPG) (4 mg in 1 mL Z buffer) was added to each well. LacZ activity was determined by following the conversion of ONPG to ortho-nitrophenol at 420 nm.

### Selection of pyrithiamine-resistant *K. pneumoniae* strains

Pyrithiamine-resistant *K. pneumoniae* strains were selected using the previously described method (16). Overnight cultures of *K. pneumoniae* were diluted 1/100 in 100 µL M9 in a transparent 96-well plate (Greiner Bio-One GmbH, Frickenhausen, Germany). The cultures were either supplemented with 1 mM of pyrithiamine or an equal volume of H_2_O and incubated at 37°C and 180 rpm in a humidity chamber. Growth of the strains was monitored in a microplate reader (Tecan Spark). When the cultures reached the stationary phase, 1 µL of each culture was used to inoculate a fresh 100 µL M9 culture containing 1 mM pyrithiamine. This procedure was continued until the cultures with pyrithiamine showed the same growth rate as wild-type *K. pneumoniae* in a culture without pyrithiamine, indicating that resistors have accumulated within the culture. Pure cultures of these resistors were obtained by plating dilutions on M9 minimal agar containing 1 mM pyrithiamine. Single colonies (13) were grown, and TPP riboswitches Kp01, Kp04, Kp10, and Kp11 were analyzed by PCR and DNA sequencing.

### Determination of TPP levels in *E. coli*

Overnight cultures of *E. coli* MG1655 in M9 were treated with 10 µM thiamine or pyrithiamine and, following cultivation, the cells were collected by centrifugation. The cell pellets were washed three times using phosphate-buffered saline. Cells were then added to a bead-beating tube with PBS and processed in the MP-Bio Fastprep 5G (MP Biomedicals, Irvine, CA, USA) for 30 seconds at 6 m/s for five cycles. Samples were centrifuged at 10,000 × *g* for 5 minutes at room temperature, and the supernatants were collected. Proteins were precipitated by adding 1 volume of methanol. TPP was analyzed by derivatization and HPLC using the “Vitamin B1 in Whole Blood and Vitamin B6 in Whole Blood/Plasma HPLC reagent kit # 52052” from Chromsystems Instruments & Chemicals GmbH (Munich, Germany).

### Bioinformatic and statistical analyses

For the prediction of riboswitch secondary structures, the PASIFIC tool (23) and the ViennaRNA Web Services (24) were employed. For the prediction of the Ec01 and Kp04 riboswitch tertiary structures, AlphaFold 3 was used (25). The corresponding pLDDT values were between 70 and 90. pLDDT is a per-atom estimate of the confidence of AlphaFold 3 in a structure prediction. It uses a 0–100 scale, where higher values indicate higher confidence. A value above 90 indicates high confidence; a value below 50 indicates the corresponding part of the predicted structure is probably wrong. For the visualization of RNA secondary structures, VARNA was used (26). All statistics were performed either in Microsoft Excel 2016 or with the GraphPad Prism 9 software. A two-tailed Student’s *t*-test was performed for all data sets. First, an *F*-test was performed to examine standard deviation equality. When the *P*-value of the *F*-test was ≤0.05, a heteroscedastic *t*-test was performed; otherwise, a homoscedastic *t*-test was performed. When the *P*-value of the *t*-test was less than 0.05, the data sets were considered significantly different. A value below 0.05 was marked with one asterisk, a value below 0.01 with two asterisks, and a value below 0.001 with three asterisks.

## RESULTS

### Characterization of TPP-responsive riboswitches from ESKAPE pathogens using a dual-luciferase system

**Fig 2:**
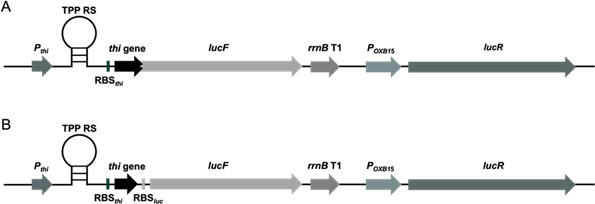
Schematic structures of the translational reporter gene test plasmid pDluc (**A**) and the transcriptional test plasmid pDlucTC (**B**). In both plasmids, expression of *lucR* encoding *Renilla* luciferase is driven by the artificial, constitutive *E. coli* promoter p_OXB15_. The *rrnB* T1 transcriptional terminator upstream of p_OXB15_ prevents readthrough from promoters, which drive expression of the riboswitches. The reporter gene *lucF* is located downstream of the first few codons of the respective thiamine biosynthesis or transport gene (*thi* gene), the corresponding ribosomal binding site (RBS_thi_), the TPP riboswitch aptamer portion (TPP-RS), and the employed promoter (e.g., p_thi_). In the translational fusion plasmid pDluc (**A**), *lucF* is directly fused to the *thi* gene codons, while in the transcriptional fusion plasmid pDlucTC (**B**), an additional ribosomal binding site (RBS_luc_) was placed between the *thi* gene and *lucF*. Binding of a ligand to the TPP riboswitch aptamer RNA triggers the downstream expression platforms, which leads to reduced gene expression either by sequestering the ribosomal binding site or by formation of a transcriptional terminator (5).

As a first step toward the use of TPP riboswitches from ESKAPE pathogens as drug targets (18), their functions as genetic control elements were validated in an *in vivo* test system based on the host *E. coli* DH5α. DNA sequences encoding putative TPP riboswitches from ESKAPE pathogens were identified in the Rfam database (27) (listed in Table S3), synthesized (Table S2), and coupled to dual-luciferase reporter plasmids. The thiamine-auxotrophic *E. coli* strain DH5α, for which 20 nM thiamine had to be added to the pre-cultures, was used as a host for this reporter system, as we were able to better control thiamine levels in this organism. The dual-luciferase reporter plasmids allowed testing of riboswitch functions and were developed in this work (Fig. 2). In the case of a “translation control” mechanism (Fig. 2A), TPP binding to the aptamer domain of the forming TPP riboswitch mRNA causes an alternative folding of this RNA, prevents ribosomes from accessing the ribosomal binding site of the reporter gene mRNAs, and thus prevents formation of the reporter protein. In the case of “transcription control” (Fig. 2B) mediated by a TPP riboswitch, binding of TPP to the nascent transcript leads to the formation of a transcriptional terminator, which in turn leads to dissociation of the RNA polymerase from the DNA template and thus termination of reporter gene expression. Transcriptional terminators and RNA secondary structures that sequester ribosomal binding sites can be predicted using bioinformatic tools (24) and, accordingly, it can tentatively be predicted whether riboswitches exert a “transcription control” or a “translation control.” In our dual-luciferase reporter assay, the firefly luciferase gene *lucF* was used to probe riboswitch activity, whereas the *Renilla reniformis* luciferase gene *lucR,* placed under the control of a constitutive promoter, served for normalization. For the initial characterization of the putative TPP riboswitches, it was important to ensure that no active TPP riboswitch sequence was overlooked, and thus two different constructs were generated for each TPP riboswitch DNA using the two different reporter plasmids. In pDluc (Fig. S1), the (predicted) ribosomal binding sites of the TPP riboswitches to be tested from ESKAPE pathogens replaced the ribosomal binding site of *lucF*, while in pDlucTC (Fig. S2), the ribosomal binding site of *lucF* was conserved. If the putative riboswitch reduced gene expression upon ligand treatment employing pDluc, control may have occurred at both the transcriptional and translational levels. If the putative riboswitch reduced gene expression upon ligand treatment employing pDlucTC, control occurred mainly at the transcription level.

**Fig 3:**
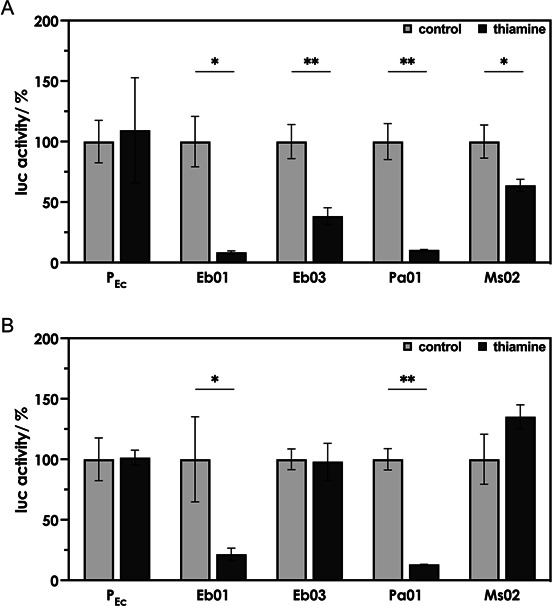
Responses of putative TPP riboswitches from pathogenic bacteria to thiamine when tested using translational (**A**) or transcriptional fusions (**B**) in combination with an *Escherichia coli* promoter. *E. coli* DH5α strains containing the dual-luciferase reporter plasmid pDluc (to test for translation control, **A**) or pDlucTC (to test for transcription control, **B**) containing putative TPP riboswitches from *Enterobacte*r spp. (*thiC*, Eb01; *thiBPQ*, Eb03), *P. aeruginosa* (*thiC*, Pa01), and *M. sciuri* (*thiE*, Ms02) were grown in M9 minimal medium in the absence (control) or presence of 10 µM thiamine. The putative TPP riboswitches were coupled to the reporter gene *lucF* and tested in combination with the *E. coli thiC* promoter P_Ec_. The control strain (P_Ec_) contained pDluc in which expression of *lucF* was solely driven by the *E. coli thiC* promoter in the absence of a TPP riboswitch. Firefly luciferase (LucF) activity was normalized to constitutive *Renilla* luciferase (LucR) activity and is shown as relative activity compared to the control. Cultures were grown in triplicate in a 12-well plate. Depicted are the mean values ± standard deviations of the data obtained from triplicate. Asterisks indicate statistically significant differences (*P ≤ 0.05, **P ≤ 0.01, and ***P ≤ 0.001).

**Fig 4:**
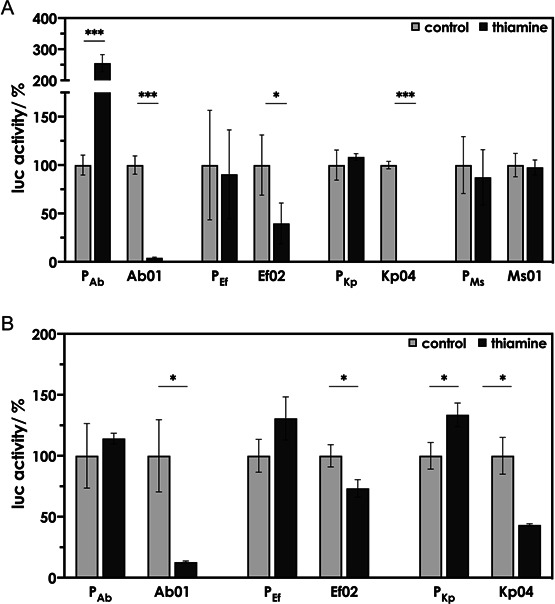
Responses of putative TPP riboswitches from pathogenic bacteria to thiamine when tested using translational (**A**) or transcriptional fusions (**B**) in combination with their native promoters. *E. coli* DH5α strains containing the dual-luciferase reporter plasmid pDluc (to test for translation control, **A**) or pDlucTC (to test for transcription control, **B**) and the putative TPP riboswitches from *Acinetobacter baumannii* (*thiC*, Ab01), *Enterococcus faecium* (*thiT*, Ef02), *Klebsiella pneumoniae* (*thiC*, Kp04), and *Mammaliicoccus sciuri* (*tenA*, Ms01) were grown in M9 in the absence (control) or presence of 10 µM thiamine. The putative TPP riboswitches were coupled to the reporter gene *lucF* and tested in combination with their native promoters P_Ab_, P_Ef_, P_Kp_, and P_Ms_. The control strains contained pDluc in which expression of *lucF* was solely driven by P_Ab_, P_Ef_, P_Kp_, and P_Ms_ in the absence of a TPP riboswitch. In the case of P_Ef_, P_Kp_, and P_Ms_, expression was not affected upon the addition of thiamine. Surprisingly, expression of *lucF* driven solely by P_Ab_ was strongly enhanced upon the addition of thiamine. Apparently, there is an unknown factor in *E. coli,* which, in combination with P_Ab_ and thiamine, stimulates LucF synthesis. Firefly luciferase (LucF) activity was normalized to constitutive *Renilla* luciferase (LucR) activity and is shown as relative activity compared to the control. Cultures were grown in triplicate in a 12-well plate. Depicted are the mean values ± standard deviations of the data obtained from triplicate. Asterisks indicate statistically significant differences (*P ≤ 0.05 and ***P ≤ 0.001).

When the putative TPP riboswitch sequences found upstream of *Enterobacter* spp. (683) *thiC* (Eb01) and *thiBPQ* (Eb03) (28), *Pseudomonas aeruginosa thiC* (Pa01), and the animal bacterial pathogen *Mammaliicoccus sciuri thiC* (Ms02) were placed downstream of the *E. coli thiC* promoter controlling the expression of the reporter *lucF* in pDluc, addition of thiamine resulted in a significant decrease in luciferase activity (Fig. 3A). No such effect was observed when the putative control elements were absent, and we conclude that the different sequences indeed function as TPP riboswitches. The experiments described above were performed using pDluc to measure possible translation control. The experiments were repeated employing pDlucTC to measure possible transcription control (Fig. 3B). In the case of Eb03, a reduction in reporter gene activity was not observed when using pDlucTC. Only pDluc allowed measuring a reduction in reporter gene activity upon challenging the riboswitches with thiamine (Fig. 3A), and we suggest that Eb03 mainly regulates at the translation level. When the putative *thiC* riboswitch sequences from *A. baumannii* (Ab01), *K. pneumoniae* (Kp04), and the *tenA* riboswitch sequence from *M. sciuri* (Ms01) were tested in combination with the *E. coli thiC* promoter using pDluc (translation control), no or only a small reduction in reporter gene activity was observed upon challenging the cultures with thiamine (Fig. S3). To further examine these sequences for a potential function as genetic switches, Ab01, Kp04, and Ms01 were also tested in our dual-luciferase reporter system in combination with their respective native promoters, P_Ab_, P_Kp_, and P_Ms_. In addition, the putative *thiT* riboswitch from *E. faecium* (Ef02) was included to be evaluated in combination with its native promoter P_Ef_, since, for unknown reasons, the coupling of the Ef02 sequence to the *E. coli* P_Ec_ promoter in pDluc was not successful. When Ab01 and Kp04 were tested in our *E. coli*-based reporter gene system employing their native (putative) upstream promoter regions, addition of thiamine led to a significantly reduced luciferase activity regardless of whether pDluc or pDlucTC was used for the analysis (Fig. 4). Even in combination with its native promoter, Ef02 was found to only weakly respond to thiamine when tested with pDluc (testing for translation control) (Fig. 4A), as well as pDlucTC (testing for transcription control) (Fig. 4B). Ms01, on the other hand, did not respond to thiamine at all when the putative promoter and riboswitch region were tested using pDluc (Fig. 4A). Due to this lack of response, no test for transcription control with pDlucTC was performed for the Ms01 sequence. Table 1 summarizes the data obtained and provides an overview of the TPP riboswitches of ESKAPE pathogens and *M. sciuri*. When the putative *thiBPQ* TPP riboswitch from *S. aureus* Sa02 was tested in combination with the *E. coli thiC* promoter, no reporter gene activity was detected (Fig. S4). Test plasmids containing the native Sa02 promoter could not be constructed. The cloning of promoter regions using high-copy plasmids was reported to be toxic for the host cell and could be an explanation for this (29). Although the riboswitches Ms01 and Ms02 of *M. sciuri* were erroneously annotated in the Rfam database as TPP riboswitches of *S. aureus*, they were nevertheless included in the present study. In our test system, Ms01 and Ms02 responded only weakly, and it is questionable whether they represent riboswitches or whether these sequences have a different function.

**TABLE 1:** Thiamine pyrophosphate riboswitches of ESKAPE pathogens and *Mammaliicoccus sciuri^a^*

Organism	RS name	Gene	Predicted	Experimental result
*Acinetobacter baumannii*	Ab01	*thiC*	Transcriptional	Translational/transcriptional
*Enterobacter* spp.	Eb01	*thiC*	Transcriptional	Transcriptional
*Enterobacter* spp.	Eb03	*thiBPQ* operon	Translational	Translational
*Enterococcus faecium*	Ef02	*thiT*	Translational	Translational/transcriptional
*Klebsiella pneumoniae*	Kp04	*thiC*	Transcriptional	Translational/transcriptional
*Pseudomonas aeruginosa*	Pa01	*thiC*	Translational	Transcriptional
*Mammaliicoccus sciuri*	Ms01	*tenA*	Transcriptional	Weak or no reaction
*Mammaliicoccus sciuri*	Ms02	*thiE* operon	Transcriptional	Weak or no reaction

^*a*^ The *in silico* predicted mode of action is compared to the experimental results.

### TPP riboswitches from ESKAPE bacteria do not respond to the antimicrobial compound pyrithiamine

**Fig 5:**
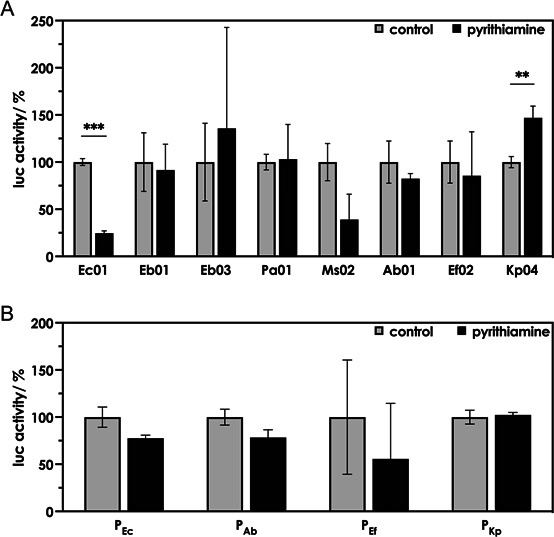
The addition of pyrithiamine does not negatively affect the activity of TPP riboswitches from different pathogenic bacteria. TPP riboswitches from *Enterobacte*r spp. (*thiC*, Eb01; *thiBPQ*, Eb03), *P. aeruginosa* (*thiC*, Pa01), and *M. sciuri* (*thiE*, Ms02) under the control of the *E. coli thiC* promoter P_Ec_ and TPP riboswitches from *A. baumannii* (*thiC*, Ab01), *E. faecium* (*thiT*, Ef02), and *K. pneumoniae* (*thiC*, Kp04) under the control of their native promoters (**A**), as well as the corresponding promoter control regions P_Ec_, P_Ab_, P_Ef_, and P_Kp_ (**B**) were coupled to pDluc, and the corresponding *E. coli* MG1655 test strains were challenged with 10 µM pyrithiamine. The controls were grown in the absence of pyrithiamine. Firefly luciferase (LucF) activity was normalized to constitutive *Renilla* luciferase (LucR) activity and is shown as relative activity compared to the controls. Cultures were grown in triplicate in a 12-well plate. Depicted are the mean values ± standard deviations of the data obtained from the triplicate. Asterisks indicate statistically significant differences (*P ≤ 0.05, **P ≤ 0.01, and ***P ≤ 0.001). The *E. coli* TPP riboswitch Ec01 was included as a control, as it was known that this riboswitch was negatively affected by pyrithiamine. Ms02 responds weakly to pyrithiamine.

The synthetic structural thiamine analog pyrithiamine reduces the growth of *E. coli* and *B. subtilis,* and the TPP riboswitches present in these bacteria were identified as targets for this antibiotic (16). Following uptake of thiamine by *E. coli*, cytoplasmic thiamine kinase (EC 2.7.1.89) and thiamine-monophosphate kinase (EC 2.7.4.16) generate TPP (20), and both kinases in concert also generate PTPP from pyrithiamine following uptake of this chemotherapeutic agent (mediated most likely by the thiamine uptake system) (30) (Fig. 1). A significant decrease in reporter gene activity was observed for the *E. coli thiC* riboswitch Ec01 coupled to its native promoter and *lucF* employing pDluc when pyrithiamine (10 µM) was added to cultures of thiamine-prototrophic *E. coli* MG1655 (Fig. 5). This pyrithiamine level was reported earlier to effectively trigger the *E. coli thiC* riboswitch (16). Notably, when the thiamine-auxotrophic *E. coli* strain DH5α, for which 20 nM thiamine had to be added to the cultures, was used as a host for these experiments, luciferase activity did not change upon addition of pyrithiamine. The presence of thiamine apparently counteracted the effect of pyrithiamine. Therefore, all the following assays were carried out using thiamine-prototrophic *E. coli* MG1655. A significant effect of pyrithiamine (but not of the natural thiamine analog bacimethrin, Fig. S5) on luciferase activity was now observed when testing Ec01 (Fig. 5). Reporter gene activity was also surprisingly reduced when testing the *M. sciuri* sequence, although this putative element only weakly responded to thiamine. In contrast, the TPP riboswitches from *Enterobacter* spp. (Eb01 and Eb03), *P. aeruginosa* (Pa01), *A. baumannii* (Ab01), and *E. faecium* (Ef02) were not negatively affected by pyrithiamine in combination with their native promoters. Interestingly, for the *K. pneumoniae* (Kp04) riboswitch, a significant increase in reporter gene activity was observed, indicating that pyrithiamine positively affected this riboswitch. The activity of Kp04 was also tested using a translational fusion plasmid (pHA191) employing *lacZ* as a reporter gene fused to the first few codons of the native downstream gene *thiC*. When the corresponding *E. coli* test strain was challenged with thiamine, a decrease in *lacZ* expression was observed (Fig. S6). This effect was absent when only the Kp04 promoter (pKp04) was present. In the next step, *K. pneumoniae* Kp04 was challenged with a series of synthetic thiamine analogs, which were commercially available (Fig. S7). None of the 18 small compounds led to a significant decrease in *lacZ* expression (Fig. S6), indicating that Kp04 was not negatively affected by these putative ligands. Interestingly, some compounds (e.g., compound 11) led to an increase in *lacZ* activity, suggesting that the RBS of Kp04 was better accessible upon binding this synthetic ligand.

To verify that thiamine and pyrithiamine were taken up by *E. coli*, cell-free extracts were prepared and analyzed for their TPP levels (Table S4). When thiamine (10 µM) was added to *E. coli* MG1655, an almost ninefold increase in cellular TPP levels was observed (from 91 µg TPP/L cell-free extract to 810 µg TPP/L, normalized to the OD_600_ values of the corresponding cultures), showing that extracellular thiamine was taken up. When cultures were treated with pyrithiamine (10 µM) instead of thiamine, less cytoplasmic TPP was found (81 µg TPP/L) when compared to the untreated control (91 µg TPP/L), indicating that pyrithiamine interfered with TPP synthesis. This finding indirectly showed that pyrithiamine indeed was taken up by *E. coli* MG1655 and had the potential to also negatively affect the tested TPP riboswitches.

### Comparison of primary and tertiary structures of TPP riboswitches from *E. coli* and *K. pneumoniae* reveals minor differences

**Fig 6:**
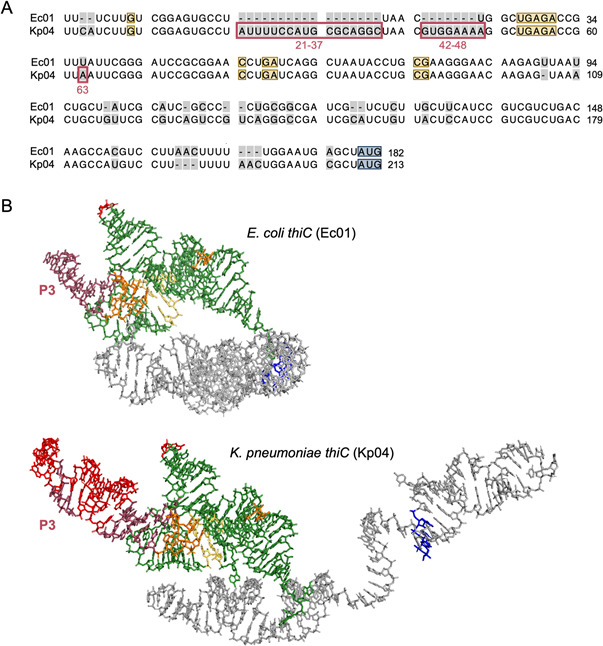
Comparison of the *thiC* TPP riboswitches from *E. coli* (Ec01) and *K. pneumoniae* (Kp04). (**A**) The DNA sequence alignment was created by employing the Needleman-Wunsch algorithm. Not identical nucleotides are highlighted in gray. Nucleotides important for TPP binding are shown in yellow. The start codon of *thiC* is highlighted in blue. The red boxes mark important differences in the aptamer regions, and numbers indicate the nucleotide position in the Kp04 sequence. (**B**) Tertiary structures of *thiC* riboswitch RNAs were predicted by AlphaFold 3 (25). The aptamer regions are colored green, and the nucleotides of the P3 stem regions are colored dark red. Nucleotides 21–37 and 42–48 of the Kp04 sequence, as well as A63 of Kp04 and the corresponding U37 of Ec01, are highlighted in bright red. The nucleotides that form the binding pockets for the aminopyrimidine ring and the pyrophosphate are highlighted in orange and yellow, respectively. The start codons of the downstream genes are boxed in blue.

TPP riboswitches of ESKAPE pathogens were not negatively affected by pyrithiamine (Fig. 5), although the aptamer regions of the riboswitches relevant for pyrithiamine binding are highly conserved. The *E. coli thiC* riboswitch responded to both thiamine and pyrithiamine, whereas the *K. pneumoniae thiC* riboswitch did not reduce reporter gene expression upon treatment with pyrithiamine (Fig. 5A). An RNA sequence alignment of the *E. coli thiC* riboswitch (Ec01) and the *K. pneumoniae thiC* riboswitch (Kp04) revealed that the primary structures of the aptamer regions of both riboswitches were very similar (Fig. 6A). Not only are the nucleotides that were predicted to form the binding pockets to accommodate the pyrimidine moiety of TPP and the pyrophosphate group identical, but the nucleotides in the immediate vicinity are also the same (Fig. 6A). However, two “insertions” (nucleotides 21–37 and 42–48) were identified in the *thiC* riboswitch sequence of *K. pneumoniae*, which were not present in the corresponding *thiC* riboswitch of *E. coli*. Moreover, the two riboswitches differ at position 63: *K. pneumoniae* has an adenosine monophosphate at this position, while *E. coli* has a uridine monophosphate. The predicted tertiary structures of the two enterobacterial riboswitches show that the two “insertions” present in the *K. pneumoniae thiC* riboswitch led to an extension of stem loop P3 (Fig. 6B). The positions of the nucleotides that were shown to be responsible for binding of pyrithiamine are not located within P3 (31, 32). Still, P3 affected the binding of pyrithiamine, as demonstrated in the following experiments.

### Single nucleotide deletions in the P3 region of the *K. pneumoniae thiC* riboswitch generate pyrithiamine-responsive TPP riboswitches

**Fig 7:**
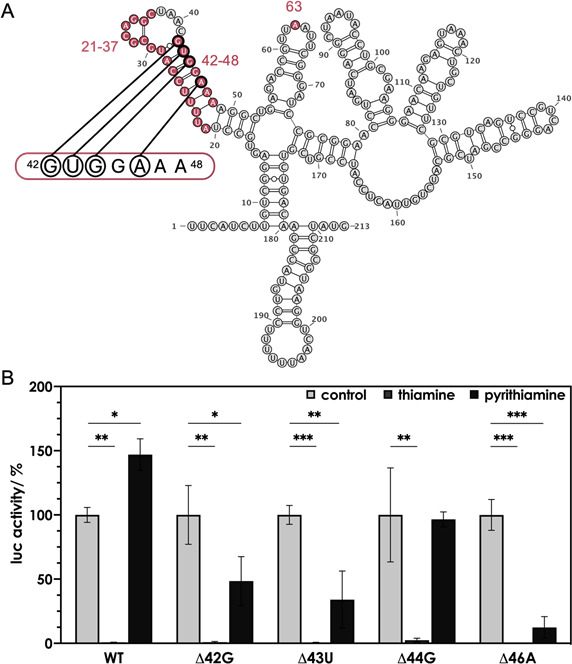
Effect of thiamine and pyrithiamine on the *K. pneumoniae thiC* Kp04 riboswitch and its variants Δ42G, Δ43U, Δ44G, and Δ46A. (**A**) The secondary structure of the Kp04 riboswitch was predicted by RNAfold (33). The red color marks nucleotides that differ from the *E. coli thiC* riboswitch sequence. Black circles indicate the deleted nucleotides from regions 42–48. (**B**) *E. coli* MG1655 pDluc::Kp04 was grown in M9 minimal medium in the absence (control) and presence of 10 µM thiamine or pyrithiamine. Firefly luciferase (LucF) activity was normalized to constitutive *Renilla* luciferase (LucR) activity and is shown as relative activity compared to the control. Cultures were grown in triplicate in a 12-well plate. Depicted are the mean values ± standard deviations of the data obtained from the triplicate. Asterisks indicate statistically significant differences (*P ≤ 0.05, **P ≤ 0.01, and ***P ≤ 0.001).

To test whether the observed sequence differences in the aptamer region of Kp04 and Ec01 (Fig. 6A) were responsible for the differences in susceptibility to pyrithiamine, the TPP riboswitch variants Kp04^A63U^, Kp04^Δ21-37^, and Kp04^Δ42-48^ were coupled to pDluc and compared to wild-type Kp04 employing our *in vivo E. coli* test system. Kp04^A63U^, Kp04^Δ21-37^, and Kp04^Δ42-48^ all responded to thiamine, i.e., addition of the Kp04 ligand thiamine to the test strains resulted in a reduction of reporter gene (*lucF*) activity. In the riboswitch variant Kp04^Δ42-48^, however, the response to thiamine was significantly reduced when compared to the wild type or the other two variants (Fig. S8). Moreover, Kp04^A63U^ and Kp04^Δ21-37^ did not respond to pyrithiamine, whereas the addition of pyrithiamine to the cultures containing the test plasmid for the Kp04^Δ42-48^ riboswitch resulted in a significant reduction of reporter gene activity (Fig. S8D). In light of these results, other Kp04 variants were constructed and tested, now focusing on the “nucleotide insertion 42–48” (Fig. 7A). The single-nucleotide deletions Δ42G, Δ43U, Δ44G, and Δ46A of Kp04 were generated and tested by employing pDluc (Fig. 7B). Notably, not all possible deletion variations were tested, e.g., deletion of 44G and deletion of 45G would result in the same primary structure of Kp04. In the case of the test strain containing Kp04^Δ44G^, no reduction in luciferase activity was observed upon addition of pyrithiamine. In contrast, Kp04 deletions 42G, 43U, and 46A led to a significant response to pyrithiamine, while maintaining the susceptibility to the similar ligand thiamine. Most interestingly, the effect on reporter activity varied in strength depending on the position of the deletion, whereby the variant Kp04^Δ46A^ responded most strongly to pyrithiamine (Fig. 7B). The following experiment was carried out to test whether the addition of the extended P3 stem of Kp04 would alter the response of the corresponding *E. coli thiC* riboswitch Ec01. The reporter plasmid pDluc::Ec01-P3 was created by inserting the extended P3 stem sequence of the *K. pneumoniae thiC* riboswitch (UUccaUgcgcaggcUaacgUggaa) between nucleotides 18 and 19 of the *E. coli thiC* riboswitch sequence Ec01 (Fig. 6A). This recombinant Ec01, however, did respond to pyrithiamine (as did wild-type Ec01) (Fig. S9), indicating that other nucleotides of the riboswitches in combination with P3 affect ligand binding.

### *K. pneumoniae* contains four TPP riboswitches that respond differently to pyrithiamine

While pyrithiamine completely inhibited the growth of *E. coli* and *B. subtilis* in liquid cultures at concentrations of 200 and 140 µM, respectively (16), addition of 300 µM pyrithiamine to a liquid culture of *K. pneumoniae* only slowed growth (Fig. S10). This finding was in line with the wild-type TPP riboswitch Kp04 not being negatively affected by pyrithiamine, while the activity of the corresponding *E. coli* TPP riboswitch Ec01 was reduced in the presence of this thiamine analog. Since *K. pneumoniae* contains more than one TPP riboswitch, we hypothesized that other TPP riboswitches present in this bacterium would be more strongly affected by pyrithiamine. Accordingly, the *K. pneumoniae* TPP riboswitches Kp01 (located upstream of the *thiBPQ* operon coding for the thiamine ABC uptake system), Kp10 (upstream of *tenA* coding for thiaminase II, EC 3.5. 99.2), and Kp11 (upstream of *thiMD* coding for bifunctional hydroxyethylthiazole kinase/phosphomethylpyrimidine kinase, ECs EC 2.7.1.50 and 2.7.4.7) were tested with regard to their responses to thiamine and pyrithiamine using pDluc. Our results show that Kp01, Kp10, and Kp11 are functional TPP riboswitches, as they all responded to the addition of thiamine to the test cultures (Fig. S11). Kp01 and Kp11 also responded to pyrithiamine, but not as strongly as to thiamine. Interestingly, Kp10 was not affected by pyrithiamine. A comparison of the riboswitch sequences gave no indication as to which of the nucleotides could be responsible for this behavior (Fig. S12). These results show that at least some TPP riboswitches are targets for pyrithiamine in *K. pneumoniae,* which explains why we observe a mild growth reduction. To further analyze *K. pneumoniae* TPP riboswitches with regard to pyrithiamine activity, resistors toward this synthetic antibiotic were isolated by stepwise challenging *K. pneumoniae* cultures with pyrithiamine until growth was similar to that of untreated cultures. In total, 13 different fully resistant *K. pneumoniae* strains were obtained. The DNA sequences encoding Kp01, Kp04, Kp10, and Kp11 (including the promoters) were amplified by PCR and sequenced. We did not find mutations in promoter sequences that would alter expression levels and allow us to overcome negative regulation by PTPP. We also did not find mutations in riboswitch sequences, and we concluded that these RNA molecules were not the primary targets for pyrithiamine.

## DISCUSSION

RNA molecules play key roles in biological processes and thus diseases. One way to develop innovative drugs is, therefore, to design small molecules that bind to RNA and affect downstream biological functions. To drive the field of RNA-targeted drug discovery forward, fundamental knowledge must be generated on how to obtain potent RNA-specific drug-like molecules, which, in turn, requires a better understanding of which RNAs are best suited to bind drug-like ligands with high affinity, i.e., are “druggable” (34). Riboswitches are RNA molecules and given the essential role of riboswitch-mediated gene regulation in bacterial physiology, they were considered potential antibiotic targets (17). Putative TPP riboswitches have been predicted in various pathogenic bacteria through the use of bioinformatic methods, including pathogens of the ESKAPE group (18, 35, 36). We initiated this work to find out whether TPP riboswitches of ESKAPE pathogens were, in principle, targets for small molecule drugs that bind to riboswitch RNAs and block downstream gene expression. This would either stop thiamine biosynthesis or thiamine transport. The lack of thiamine-containing cofactors in the cell reduces the activity of enzymes that require these cofactors for their activity (37). As a result, the corresponding biochemical processes are either slowed down or come to a complete standstill, which ultimately reduces the growth of the target bacteria.

In the present study, a dual-luciferase reporter gene assay providing the advantage of normalization with regard to plasmid copy numbers was developed to study TPP riboswitch functions *in vivo*. We found that not all putative TPP riboswitch sequences identified by bioinformatic tools indeed responded to thiamine using our test system. Second, as expected, the use of different promoters of varying strength affected riboswitch function (38). Finally, the use of different reporter plasmids suggested different modes of action for the characterized riboswitches (Table 1) (5, 39), although we cannot rule out the possibility that the activity of the analyzed putative riboswitch sequences depended on yet other factors or was driven by another mechanism. Our main goal was not to describe the TPP riboswitches mechanistically. The reason why we used both transcriptional and translational fusions of the TPP riboswitches was that we did not want to miss any functional riboswitch sequence. The fact that a reduction in luciferase activity upon thiamine treatment was observed for Kp04 using pDlucTC (Fig. 3B) as well as pDluc (Fig. 3A) suggests a combination of transcriptional and translational control for this riboswitch. It demonstrates that it is challenging to predict and also study the mechanism of action of these highly dynamic regulatory elements, which build up at a speed of about 40–80 nucleotides per second.

Small molecule compounds targeting TPP riboswitches have so far only been tested on already characterized TPP riboswitches (10, 40, 41). The present work on TPP riboswitches of ESKAPE pathogens significantly extended this concept and revealed that none of the riboswitches exhibited a response to the synthetic thiamine analog pyrithiamine. An exemplary in-depth comparison of the *E. coli* and *K. pneumoniae thiC* riboswitches demonstrated that a single-nucleotide deletion within the riboswitch sequence maintains the riboswitch responsiveness to the natural ligand TPP, while changing the susceptibility to other ligands such as pyrithiamine pyrophosphate. This implies that a single nucleotide insertion can render the riboswitch resistant to other compounds while maintaining its affinity for the natural ligand. This is of particular interest, given that previous studies have reported that single nucleotide changes within the riboswitch sequence typically result in the elimination of a response to both TPP and pyrithiamine (16, 31). We show that specific nucleotides of the extended P3 stem of the *K. pneumoniae* TPP riboswitch Kp04 appear to play a role in discriminating between similar ligands, although the ligands bind the riboswitch RNA at a different site. Interestingly, extended P3 stems are present in TPP riboswitches from *Klebsiella aerogenes* and several *Serratia* species (39), and a considerable diversity in length and sequence of the P3 stem has also been described for eukaryotic (plant) riboswitches (42, 43). It was further reported that, despite not being directly involved in ligand binding (31, 32), the distal part of P3 of the *Arabidopsis thaliana* TPP-sensing *thiC* riboswitch might act as an anchor for the aptamer (44, 45). Unfortunately, co-crystallization studies on this riboswitch bound to TPP or PTPP were carried out with a riboswitch in which P3 was shortened for crystallization (31, 32). Whether or not an extended P3 stem is involved in the binding of PTPP, therefore, remains unclear. In contrast to what we expected, the *K. pneumoniae* Kp04 riboswitch was positively affected by pyrithiamine, i.e., reporter gene expression was stimulated upon adding pyrithiamine to the test strain. This was only observed when the native promoter of Kp04 was used to drive reporter gene expression. When the stronger *E. coli thiC* promoter was used, pyrithiamine did not affect Kp04 (Fig. S13). We assume that the TPP analog PTPP present in the cytoplasm of pyrithiamine-treated *E. coli* cells (Fig. 1) is the actual effector of the riboswitch, which binds to the aptamer RNA and leads to stabilization of a structure that favors exposure of the ribosomal binding site.

The fact that pyrithiamine was not active against TPP riboswitches of ESKAPE bacteria does not mean that TPP riboswitches of these organisms, in principle, are not “druggable.” In the literature, we find an example of a successful screening program that led to the identification of ribocil, which targets FMN riboswitches of a variety of bacteria, including methicillin-resistant *Staphylococcus aureus* and *Enterococcus faecalis,* and reduces downstream gene expression of riboflavin biosynthesis and transport genes (46, 47). The natural riboflavin analog or antivitamin roseoflavin (48) has a similar effect and inhibits FMN riboswitches highly effectively (22). Synthetic ribocil binds to the aptamer portion of FMN riboswitches, which have evolved to bind FMN at very low levels (6). However, in contrast to roseoflavin, which is converted intracellularly to the FMN analog RoFMN and inhibits FMN-dependent flavoenzymes (49), ribocil apparently has no such negative effects on these proteins.

In the case of TPP riboswitches from ESKAPE bacteria, we have shown using an *in vivo* model that these genetic elements are affected by thiamine, in a few cases by pyrithiamine, and also by other thiamine analogs. Interestingly, our very small screening program identified a compound that affected riboswitch activity in a positive way. Antivitamins such as pyrithiamine were developed decades ago to probe enzymes when it was realized that vitamins and their cofactor derivatives play an important role in metabolism and could be useful to slow down cancer cell growth. Of course, the idea came up to use these vitamin analogs also to inhibit bacterial or fungal growth (50). At that time, riboswitches were not yet known as targets for vitamins or cofactor derivatives. Natural thiamine analogs that block TPP riboswitches are not known, but it is very likely that they exist. Notably, bacimethrin, a natural product isolated from *Bacillus megaterium* and *Streptomyces albus*, is converted to the TPP analog 2′-methoxy-thiamine pyrophosphate. Of the seven thiamine pyrophosphate-utilizing enzymes in *E. coli*, 2′-methoxy-thiamine pyrophosphate inhibits three of them (51). Based on structural information about riboswitches, it is now possible to develop synthetic compounds that could be useful as anti-infectives. An obvious approach is to screen large compound libraries (as in the case of ribocil), and we are highly confident that a ligand can be found that negatively affects TPP riboswitches at low concentrations (18).

## SUPPLEMENTAL MATERIAL

Supplemental material jb.00308-25-s0001.docx Tables S1 to S4 and Figures S1 to S13.

ASM does not own the copyrights to Supplemental Material that may be linked to, or accessed through, an article. The authors have granted ASM a non-exclusive, world-wide license to publish the Supplemental Material files. Please contact the corresponding author directly for reuse.
