# Supplementary material for: Functional analysis of thiamine pyrophosphate-responsive riboswitches in human bacterial pathogens of the ESKAPE group using a dual-luciferase reporter gene assay
Source: J Bacteriol. 2025 Oct 9;207(11):e00308-25. doi: 10.1128/jb.00308-25 (PMC12632268; doi:10.1128/jb.00308-25)
Supplement: Supplemental material — Tables S1 to S4 and Figures S1 to S13. [file jb.00308-25-s0001.docx]

**Functional analysis of thiamine pyrophosphate-responsive riboswitches in human bacterial pathogens of the ESKAPE group using a dual-luciferase reporter gene assay**

Anna Hübenthal^1^, Vipul Panchal^2^, Ruth Brenk^2^ and Matthias Mack^1*^

^*^Corresponding author.

^1^Institute for Technical Microbiology, Department of Biotechnology, Hochschule Mannheim, 68163 Mannheim, Germany

^2^Department of Biomedicine, University of Bergen, 5020 Bergen, Norway

**Contact**

**^*^**Address correspondence to:

Matthias Mack, Institute for Technical Microbiology, Hochschule Mannheim, Paul-Wittsack-Str. 10, 68163 Mannheim, Germany, Phone:+49-621-292-6496, Fax:+49-621-292-6420, E-mail: m.mack@hs-mannheim.de.

# Supplemental Tables and Figures

**Table S1: Oligonucleotides used in this study.**

| **Oligonucleotide** | **Sequence (5’-3’)** | **Application** | | |
| --- | --- | --- | --- | --- |
| pRib-luc_fwd | AGCTATTGTAATCCTCCGAG | Sequencing/PCR for OXB15-*luc^F^* in pluc plasmid | | |
| pRib-luc_rev | GGTGATGCCAATTCGGTT |  |  |  |
| pluc_seq | GAGTCAGTGAGCGAGGAA | Sequencing/PCR for pDluc/pDlucTC plasmids | | |
| pluc_rev | TTTGGCGTCTTCTGCTTG |  |  |  |
| pluc_TC_rev | CCAGGGCGTATCTCTTCA |  |  |  |
| *rrnB*T1_*Sac*I_fwd | ^a^TACAT^b^**GAGCT**CTAGGGAACTGCCAGGCATCA | Amplification of *rrnB*T1 for pPrib-RFN-luc.t at *Sac*I site, additionally inserting *Xho*I site | | |
| *rrnB*T1_*Sac*I_*Xho*I_rev | TACAT**GAGCTC**CAGAA**CTCGAG**TACTCAGGAGAGCGTT |  |  |  |
| Pluc_*Kpn*I-ins_fwd | ATAGGTC**GGTACC**GAGCTCCCAAAAAAAAAA | Insertion of *Kpn*I site between *Xho*I and *Sac*I site | | |
| Pluc_*Kpn*I-ins_rev | **GGTACC**GACCTATCAGAACTCGAGTACTCAG |  |  |  |
| OXB15-luc^R^_fwd | TCAGA**CTCGAG**TACTTGT | Amplification of OXB15-luc^R^ fragment with *Xho*I & *Kpn*I restriction sites | | |
| OXB15-luc^R^_*Kpn*I_rev | TCGTA**GGTACC**TGTCA**CTGCAG**TTACTGTT |  |  |  |
| *Nco*I_ins_fwd | GCTAATCATTAGCGTTATAGTGAATCCGCT**CCATGG**TTTAAGGACAAATGAATAAAGATTGTATCC | Replacing the *Hin*dIII restriction site of pPrib-Dluc with an *Nco*I site | | |
| *Nco*I_ins_rev | GGATACAATCTTTATTCATTTGTCCTTAAA**CCATGG**AGCGGATTCACTATAACGCTAATGATTAGC |  |  |  |
| pDluc_RS-del_fwd | CATGGGGGAGGGAAACAAATGTG | Deletion of FMN riboswitch from pPrib-RFN-Dluc | | |
| pDluc_RS-del_rev | GATCCACATTTGTTTCCCTCCCC |  |  |  |
| pDluc_Δprom_fwd | CTGTGCTGCCCAAGGTATATCTCC | Deletion of T7 promoter in pT7Dluc | | |
| pDluc_Δprom_rev | CATGGGAGATATACCTTGGGCAGCACAG |  |  |  |
| pDluc_RBSins_fwd | AAACAAATGTGGATCCAATAGAGGGCCCGCATCCAAATGGAAGACGCCAAAAAC | Insertion of RBS for luc^F^ to create transcriptional fusion plasmid pDlucTC | | |
| pDluc_RBSins_rev | GTTTTTGGCGTCTTCCATTTGGATGCGGGCCCTCTATTGGATCCACATTTGTTT |  |  |  |
| Ec01_fwd | TCACT**CAGCTG**GCTGGAAGATAAGCTGATT | Amplification of *E. coli* *thiC* RS region |  | |
| Ec01_rev | ACTTCGTCTC**GGATCC**AGCGTTGTTCGCGGCGGGT |  | Amplification of *E. coli* *thiC* promoter region | |
| pEc01_pluc_rev | TCACT**CCATGG**GGGGCATTGAATGTAAAT |  |  |  |
| Ab01_fwd | TACAT**CCATGG**TTAAATCGCTTGACGGAG | Amplification of *A. baumannii thiC* RS region |  | |
| Ab01_rev | AACAT**GGATCC**AAGAGAGATTCGTTAATTG |  | Amplification of *A. baumannii thiC* prom+RS region | |
| Ab01_prom_fwd | TCACT**CAGCTG**GTATAGATACAAATTGAC |  |  |  |
| Ef02_prom_fwd | TAT**CAGCTG**CCAGTTAGTCCATTGTTTG | Amplification of *E. faecium* ABC transp. prom+RS region | | |
| Ef02_rev | TACAT**GGATCC**GTTCTACCCAAACTCTATT |  |  |  |
| Kp04_fwd | TACAT**CCATGG**TTTCATCTTGTCGGAGTG | Amplification of *K. pneumoniae thiC* RS region | |  |
| Kp04_rev | ACTTCGTCTC**GGATCC**AACGGGTTAGTTTGGTAGT |  |  | Amplification of *K. pneumoniae thiC* prom+RS region |
| Kp04_prom_fwd | ATCAT**CAGCTG**GATAATATTGCGCCGACC |  | |  |
| Eb01_fwd | TACAT**CCATGG**AAATTTCTTGTCGGAGTGC | Amplification of *Enterobacter* spp*. thiC* RS region | | |
| Eb01_rev | ATCGTCTC**GGATCC**AGCGGCGGGTCAATTTTGC |  |  |  |
| Eb03_fwd | TACAT**CCATGG**GCTGTTCTCAACGGGGTG | Amplification of *Enterobacter* spp*. thiBPQ* RS region | | |
| Eb03_rev | TACAT**GGATCC**TCGCCAGCAGGGGGAGAAC |  |  |  |
| Ms01_fwd | GAGA**CCATGG**TTAAACCACTGGAAGTGC | Amplification of *M. sciuri tenA* RS region | |  |
| Ms01_rev | CACA**GGATCC**GTTATAATTAAAAAAACGC |  |  | Amplification of *M. sciuri tenA* prom+RS region |
| Ms01_prom_fwd | CGT**CAGCTG**ATTTTATTTGCAATCCTAG |  | |  |
| Ms02_fwd | TATA**CCATGG**AATGTCCACTGGAAGTGC | Amplification of *M. sciuri thiE* RS region | | |
| Ms02_rev | GACGA**GGATCC**ATTTATACGGTGTAACAGC |  |  |  |
| Pa01_fwd | TATA**CCATGG**GGGTTCTTGTCGGGGTGC | Amplification of *P. aeruginosa thiC* RS region | | |
| Pa01_rev | TACGTCTC**GGATCC**CGCGGATGTCCGGGCGCGA |  |  |  |
| Sa02_fwd | TATA**CCATGG**AAATCGCACACACTAGGG | Amplification of *S. aureus* *thiBPQ* RS region | | |
| Sa02_rev | TATA**GGATCC**GTTCAGATAGCTTTAAACC |  |  |  |
| Kp01_prom_fwd | TAT**CAGCTG**CATGGTTTGCGC | Amplification of *K. pneumoniae thiBPQ* prom+RS region | | |
| Kp01_rev | TATA**GGATCC**ACGCCAGCA |  |  |  |
| Kp10_prom_fwd | ATA**CAGCTG**TTCTTGCGC | Amplification of *K. pneumoniae tenA* prom+RS region | | |
| Kp10_rev | ATA**GGATCC**AGTAAAGACCTTG |  |  |  |
| Kp11_prom_fwd | CAT**CAGCTG**GGCCGCGAA | Amplification of *K. pneumoniae thiM* prom+RS region | | |
| Kp11_rev | TATA**GGATCC**AGACAGGCGC |  |  |  |
| Kp04_A63U_fwd | AGGCTGAGACCGTTtATTCGGGATCCGCG | Site-directed mutagenesis of *K. pneumoniae thiC* RS (change A63 to T) | | |
| Kp04_A63U_rev | CGCGGATCCCGAAT^c^aAACGGTCTCAGCCT |  |  |  |
| Kp04_del21-37_fwd | ATCTTGTCGGAGTGCCTTAACGTGGAAAAGGCTG | Site-directed mutagenesis of *K. pneumoniae thiC* RS (deletion of nucleotides 21-37) | | |
| Kp04_del21-37_rev | CAGCCTTTTCCACGTTAAGGCACTCCGACAAGAT |  |  |  |
| Kp04_del42-48_fwd | CATGCGCAGGCTAACAGGCTGAGACCGTTA | Site-directed mutagenesis of *K. pneumoniae thiC* RS (deletion of nucleotides 42-48) | | |
| Kp04_del42-48_rev | TAACGGTCTCAGCCTGTTAGCCTGCGCATG |  |  |  |
| Kp04_del42G_fwd | CCATGCGCAGGCTAACTGGAAAAGGCTGAG | Site-directed mutagenesis of *K. pneumoniae thiC* RS (deletion of 42G) | | |
| Kp04_del42G_rev | CTCAGCCTTTTCCAGTTAGCCTGCGCATGG |  |  |  |
| Kp04_del43U_fwd | ATGCGCAGGCTAACGGGAAAAGGCTGAGAC | Site-directed mutagenesis of *K. pneumoniae thiC* RS (deletion of 43U) | | |
| Kp04_del43U_rev | GTCTCAGCCTTTTCCCGTTAGCCTGCGCAT |  |  |  |
| Kp04_del44G_fwd | TGCGCAGGCTAACGTGAAAAGGCTGAGACC | Site-directed mutagenesis of *K. pneumoniae thiC* RS (deletion of 44G) | | |
| Kp04_del44G_rev | GGTCTCAGCCTTTTCACGTTAGCCTGCGCA |  |  |  |
| Kp04_del46A_fwd | GCAGGCTAACGTGGAAAGGCTGAGACCG | Site-directed mutagenesis of *K. pneumoniae thiC* RS (deletion of 46A) | | |
| Kp04_del46A_rev | CGGTCTCAGCCTTTCCACGTTAGCCTGC |  |  |  |
| Kp01_gen_fwd | GCTGGTTCGATTTTAAATCC | Amplification of the Kp01 RS region of *K. pneumoniae* | | |
| Kp01_gen_rev | CATAGGTATAGACGGTAAGA |  |  |  |
| Kp04_gen_fwd | ATAACCTGAGCAATGACGTG | Amplification of the Kp04 RS region of *K. pneumoniae* | | |
| Kp04_gen_rev | GTGAATTAGGAAAAGCGGTA |  |  |  |
| Kp10_gen_fwd | CGCCGACAAAGAAACTATTG | Amplification of the Kp10 RS region of *K. pneumoniae* | | |
| Kp10_gen_rev | TCAGATAGCGACGAAAGGC |  |  |  |
| Kp11_gen_fwd | AATGCAATCCCGTCCGGTC | Amplification of the Kp11 RS region of *K. pneumoniae* | | |
| Kp11_gen_rev | CAGAACATTGGCGGTAAAGG |  |  |  |
| Ec01-P3_fwd | CTTGTCGGAGTGCCTTTTCCATGCGCAGGCTAACGTGGAAAACTGGCTGAGACCG | Site-directed mutagenesis of *E. coli* *thiC* riboswitch (insertion of P3 stem sequence) | | |
| Ec01-P3_rev | CGGTCTCAGCCAGTTTTCCACGTTAGCCTGCGCATGGAAAAGGCACTCCGACAAG |  |  |  |

^a^Overhangs are underlined, ^b^recognition sites for restriction enzymes are depicted in bold. ^c^Lower-case letters indicate insertions/nucleotide changes for site-directed mutagenesis.

Table S2: DNA sequences encoding control sequences (e.g. pEc01 containing a promoter only) and TPP riboswitches (e.g. Ec01 containing a promoter and a downstream regulatory element) from different bacteria were coupled to the test vectors pDluc and pDlucTC.

| **pEc01** | **^a^CAGCTG**GCTGGAAGA**^b^**TAAGCTGATTCTGCTGGTGCTTGACGCCGCCCGCGTCAAACATCCTGCTTGAGTTCTGCGCTGTTAACGCGTAATTTACATTCAATGCCCC**^a^CCATGG**GGG**^d^**AGGGAAACAA**^e^**ATGT**^a^GGATCC** |
| --- | --- |
| **Ec01** | **CAGCTG**GCTGGAAGATAAGCTGATTCTGCTGGTGCTTGACGCCGCCCGCGTCAAACATCCTGCTTGAGTTCTGCGCTGTTAACGCGTAATTTACATTC**^c^**AATGCCCCATTTGCGGGGCTAATTTCTTGTCGGAGTGCCTTAACTGGCTGAGACCGTTTATTCGGGATCCGCGGAACCTGATCAGGCTAATACCTGCGAAGGGAACAAGAGTTAATCTGCTATCGCATCGCCCCTGCGGCGATCGTCTCTTGCTTCATCCGTCGTCTGACAAGCCACGTCCTTAACTTTTTGGAATGAGCT**^f^**ATGTCTGCAACAAAACTGACCCGCCGCGAACAACGCT**GGATCC** |
| **pEc01-Ab01** | **CAGCTG**GCTGGAAGATAAGCTGATTCTGCTGGTGCTTGACGCCGCCCGCGTCAAACATCCTGCTTGAGTTCTGCGCTGTTAACGCGTAATTTACATTCAATGCCCC**CCATGG**TTAAATCGCTTGACGGAGCGCGAGTAATAGCTCGCTGAGATTGTGTAAATTTCGTGTTATCGCAACACCAGTTTGATCACAAGTACCGTTGAACCTGATCAGGTTAAGACCTGCGTAGGAATCAAGCCATCTGAAAACCTAAGCCCTCAATTTATCTAGTTCAAGATAAATCCGCCATCGTTTTTGGTCGTGCTTGATTCGTTGATGTTATTCATAAGGATCATGTGATGAACCAATTAACGAATCTCTCTT**GGATCC** |
| **Ab01** | **CAGCTG**GTATAGATACAAATTGACAATGCCAGCCAACCTAAAACATGTTAGTCTTTAAATCGCTTGACGGAGCGCGAGTAATAGCTCGCTGAGATTGTGTAAATTTCGTGTTATCGCAACACCAGTTTGATCACAAGTACCGTTGAACCTGATCAGGTTAAGACCTGCGTAGGAATCAAGCCATCTGAAAACCTAAGCCCTCAATTTATCTAGTTCAAGATAAATCCGCCATCGTTTTTGGTCGTGCTTGATTCGTTGATGTTATTCATAAGGATCATGTGATGAACCAATTAACGAATCTCTCTT**GGATCC** |
| **pAc01** | **CAGCTG**GTATAGATACAAATTGACAATGCCAGCCAACCTAAAACATGTTAGTCTTTAAATCGCTTGACGGAGCGCGAGTAATA**CTGCAG**GTGCTTGATTCGTTGATGTTATTCATAAGGATCATGTGATGAACCAATTAACGAATCTCTCTT**GGATCC** |
| **pEc01-Eb01** | **CAGCTG**GCTGGAAGATAAGCTGATTCTGCTGGTGCTTGACGCCGCCCGCGTCAAACATCCTGCTTGAGTTCTGCGCTGTTAACGCGTAATTTACATTCAATGCCCC**CCATGG**AAATTTCTTGTCGGAGTGCCCAGTGCGTAAGCCGGGCTGAGACCGTTAATTCGGGATCCGCGGAACCTGATCAGGCTAATACCTGCGAAGGGAACAAGAGTCAATCTGCTGTTGTATCGCCTCTGGGCGATCACCTCTTGCTTCATCCGTCGTCTGACAAGCCACTTCCTTTACTATTTTGGAATGAGCTATGTCTGCAAAATTGACCCGCCGCA**GGATCC** |
| **pEc01-Eb03** | **CAGCTG**GCTGGAAGATAAGCTGATTCTGCTGGTGCTTGACGCCGCCCGCGTCAAACATCCTGCTTGAGTTCTGCGCTGTTAACGCGTAATTTACATTCAATGCCCC**CCATGG**GCTGTTCTCAACGGGGTGCTGCATCAACGATGTGCGCTGAGATAATACCCGTCGAACCTGATCCGGATAACGCCGGCGAAGGGATTTGAGGCTGTCGCTCAAAATCCTTTGCCACTCAACTTTGAGGTGCAAAGTGTTAAAAAAAGTTCTCCCCCTGCTGGCGA**GGATCC** |
| **Ef02** | **CAGCTG**CCAGTTAGTCCATTGTTTGAAACAAAAAATAAATAATAAATAAACTCACAAAGGGGAGTCCAATTGGGCTGAGATTGAATCTATTTCTAAACCCTTCGTACCTGTATCGGTTATGCGAGCGTAGGAATTGTGAATAAACAGCTGTTTGACTATTTTCGAAAGCTGCTTCCTCCTTTGTGACTTTTTTATTCAAAGGAGGAAGTTTTTTTATGGGAAAAAATAGAGTTTGGGTAGAAC**GGATCC** |
| **pEf02** | **CAGCTG**CCAGTTAGTCCATTGTTTGAAACAAAAAATAAATAATAAATAAACTCACAAAGGGGAGTCCAACTTCCTCCTTTGTGACTTTTTTATTCAAAGGAGGAAGTTTTTTTATGGGAAAAAATAGAGTTTGGGTAGAAC**GGATCC** |
| **Kp01** | **CAGCTG**CATGGTTTGCGCCGCCGGAGCCGTAAGCGCTTTCCGTCATCTGACAAAATCATTACACTAAGGCCGTTCTCAACGGGGTGCTAATAAACATACGCAATATCATTCAAGTTGCATCAAGGCAGCAAGCGGGTGAATCCCCTGGAGCATAGATAACTATGTGACTGGGGTGAACGCGCGAAGCTAACGCAGATGCGGCTTGAAGGATGAAGCGCATGGGCTGAGAAAATACCCGTCGAACCTGATCCGGATAACGCCGGCGAAGGGATTTGAGGCTCACTCAAAATCCTTTGCCACCCCTTTTTTCTGAGGTGCAAAGTGTTGAAAAAATTACTCCCGCTGCTGGCGT**GGATCC** |
| **pKp01** | **CAGCTG**CATGGTTTGCGCCGCCGGAGCCGTAAGCGCTTTCCGTCATCTGACAAAATCATTACACTAAGGCCG**CTGCAG**TCTGAGGTGCAAAGTGTTGAAAAAATTACTCCCGCTGCTGGCGT**GGATCC** |
| **pEc01-Kp04** | **CAGCTG**GCTGGAAGATAAGCTGATTCTGCTGGTGCTTGACGCCGCCCGCGTCAAACATCCTGCTTGAGTTCTGCGCTGTTAACGCGTAATTTACATTCAATGCCCC**CCATGG**TTTCATCTTGTCGGAGTGCCTATTTTCCATGCGCAGGCTAACGTGGAAAAGGCTGAGACCGTTAATTCGGGATCCGCGGAACCTGATCAGGCTAATACCTGCGAAGGGAACAAGAGTAAACTGCTGTTCGCGTCAGTCCGTCAGGGCCGATCGCATCTGTTACTCCATCCGTCGTCTGACAAGCCATGTCCTTTTTTAACTGGAATGCGCTATGTCTACTACCAAACTAACCCGTT**GGATCC** |
| **Kp04** | **CAGCTG**GATAATATTGCGCCGACCGCTTGAGTTGTGAACCGTTAACGCGTAATTTACGTACATTATCCCTCTGCGGAGGGATTTCATCTTGTCGGAGTGCCTATTTTCCATGCGCAGGCTAACGTGGAAAAGGCTGAGACCGTTAATTCGGGATCCGCGGAACCTGATCAGGCTAATACCTGCGAAGGGAACAAGAGTAAACTGCTGTTCGCGTCAGTCCGTCAGGGCCGATCGCATCTGTTACTCCATCCGTCGTCTGACAAGCCATGTCCTTTTTTAACTGGAATGCGCTATGTCTACTACCAAACTAACCCGTT**GGATCC** |
| **pKp04** | **CAGCTG**GATAATATTGCGCCGACCGCTTGAGTTGTGAACCGTTAACGCGTAATTTACGTACATTATCCCTCTGCGGAGGGATTTCAT**CTGCAG**GCCATGTCCTTTTTTAACTGGAATGCGCTATGTCTACTACCAAACTAACCCGTT**GGATCC** |
| **Kp10** | **CAGCTG**TTCTTGCGCGGAAAAGCGCTGGCTATCCCCCATTACCATCATCCTGCGTCAAGGTGGCGCGGTGATTGTATTCAAAAGGTGAATGTCGTATAACATAGCGCTACCGAGGGGTGTCCCGTGAGGGCTGAGATGGCGCAAGCCGAACCCTTTGAACCTGATCTGGGTCATGCCAGCGAAGGGACGGGTCGGCAATTTTGCCAGCATTACCGCCATTGCCTGTTCACACCCCTGCACGCCCGGATCTCCCCTGAATAAATGGAGGTCCTGTGATCGTTCCCGCTTTTAGCCAAGGTCTTTACT**GGATCC** |
| **pKp10** | **CAGCTG**TTCTTGCGCGGAAAAGCGCTGGCTATCCCCCATTACCATCATCCTGCGTCAAGGTGGCGCGGTGATTGTATTCAAAAGGTGAATGTCGTATAACATAGCGC**CTGCAG**AAATGGAGGTCCTGTGATCGTTCCCGCTTTTAGCCAAGGTCTTTACT**GGATCC** |
| **Kp11** | **CAGCTG**GGCCGCGAAGGTGTCGTATCCAGGGCTAAAGGCTTGTTGTATGAACCGGTGAGTTGCGGTAAATTCCACAACGCTTAACAATTTCACTATTTTGACTCGGGGTGCCCTTCTTCGTTGAAGGCTGAGAAATACCCGTACCACCTGATCTGGATAATGCCAGCGTAGGGAAGTCAGAGACCGCAGGGTCATTGCTTCTACCTCGTCTGGCGGGAGCAAACTATGCCTGAGCTGTTGAATCCCGCGCCTGTCT**GGATCC** |
| **pKp11** | **CAGCTG**GGCCGCGAAGGTGTCGTATCCAGGGCTAAAGGCTTGTTGTATGAACCGGTGAGTTGCGGTAAATTCCACAACGCTTAACAATTTCACTATTT**CTGCAG**TGGCGGGAGCAAACTATGCCTGAGCTGTTGAATCCCGCGCCTGTCT**GGATCC** |
| **pEc01-Ms01** | **CAGCTG**GCTGGAAGATAAGCTGATTCTGCTGGTGCTTGACGCCGCCCGCGTCAAACATCCTGCTTGAGTTCTGCGCTGTTAACGCGTAATTTACATTCAATGCCCC**CCATGG**TTAAACCACTGGAAGTGCCTTGATAAAGGCTGAGATTAAAGTGATACCTTTAAAATTCCTTGAACCTGATCCAGCTTATACTGGCGTAGGAAAGTGGCGTATTGATTTTAAGGTACTCATGACGCTATTTTCTTAATGAAAATAGCGTTTTTTTAATTATAAC**GGATCC** |
| **Ms01** | **CAGCTG**ATTTTATTTGCAATCCTAGGATAAAACAGATATGTTGAAAGAGTATAGAAATTAAATAATTTTAAACCACTGGAAGTGCCTTGATAAAGGCTGAGATTAAAGTGATACCTTTAAAATTCCTTGAACCTGATCCAGCTTATACTGGCGTAGGAAAGTGGCGTATTGATTTTAAGGTACTCATGACGCTATTTTCTTAATGAAAATAGCGTTTTTTTAATTATAAC**GGATCC** |
| **pMs01** | **CAGCTG**ATTTTATTTGCAATCCTAGGATAAAACAGATATGTTGAAAGAGTATAGAAATTAAATAATT**CTGCAG**TAAGGTACTCATGACGCTATTTTCTTAATGAAAATAGCGTTTTTTTAATTATAAC**GGATCC** |
| **pEc01-Ms02** | **CAGCTG**GCTGGAAGATAAGCTGATTCTGCTGGTGCTTGACGCCGCCCGCGTCAAACATCCTGCTTGAGTTCTGCGCTGTTAACGCGTAATTTACATTCAATGCCCC**CCATGG**AATGTCCACTGGAAGTGCCTTATTATAGGCTGAGACTGAAGTAGTGACTTCGGGATTCCTTGAACCTGATCCAGTTCATACTGGCGTAGGAAAGTGGCGTATATAAGCTTACTTATACTTATAATTAAATTTTTACGCTATTTTTCTATCCAGAAAAATAGCGTTTTTTATTTGGAGTGATTTCATTGTTTATTGCTGTTACACCGTATAAAT**GGATCC** |
| **pEc01-Pa01** | **CAGCTG**GCTGGAAGATAAGCTGATTCTGCTGGTGCTTGACGCCGCCCGCGTCAAACATCCTGCTTGAGTTCTGCGCTGTTAACGCGTAATTTACATTCAATGCCCC**CCATGG**GGGTTCTTGTCGGGGTGCCCTATACGAGGGGCTGAGATCGGATAGTTCCGGATCCCGTTGAACCTGATCGGGCTAGCGTCCGGTTCCGCCTCGCGCGGACCGCAGCAACCCGCGTAGGGAACAAGATGTCCGCGCCATGCCTGCCTTGCCGGCGCGTCCTTCTCGCCACCAGTCCCGGTGGCCCCGCGTGCCAGGTTCTCCCGACAATCACCCAGGAGAGCCATCGATGAGCGCAACGCAGAAGAACAACATCACCCGCCTTGAGCAGCTCGACCGCCAGTCGACGCAGCCTTTCCCGAACTCGCGCAAGGTCTACCTGACCGGCTCGCGCCCGGACATCCGCG**GGATCC** |
| **pEc02-Sa02** | **CAGCTG**GCTGGAAGATAAGCTGATTCTGCTGGTGCTTGACGCCGCCCGCGTCAAACATCCTGCTTGAGTTCTGCGCTGTTAACGCGTAATTTACATTCAATGCCCC**CCATGG**AAATCGCACACACTAGGGGTGTTTTATACTGAGATGAGGCTTGCCCTCAAACCCTTTGAACCTGATCTAGCTTGAACTAGCGTAGGAAAGTGTTACTATACATATGTTTTACTAATATATATTGTGAACGCATAACTTTCCTATGGATGGTTGTGCGTTTTTTTATTAGGAGGATGTAAAAATGTCAAAAGGTTTAAAGCTATCTGAAC**GGATCC** |

^a^Restriction sites are displayed in bold. ^b^Predicted promoter regions are highlighted in green. ^c^The TPP riboswitches are highlighted in purple. ^d^Predicted ribosomal binding sites are highlighted in grey. ^e^Start codons. **^f^**The thiamine genes (first few codons) are highlighted in blue.

Table S3: Rfam accession numbers of TPP riboswitch aptamers analyzed in this study

| **Name** | **Rfam accession number** | **Length (nucleotides)** | **Start/end of riboswitch nucleotides** |
| --- | --- | --- | --- |
| Ec01 | URS00003982CB_83333 | 101 | AAUU...UGCU |
| Ab01 | URS00002F0C47_470 | 135 | AAUC...AAAC |
| Eb01 | URS0000AB6801_399742 | 109 | AAUU...UGCU |
| Eb03 | URS0000ABAC5B_399742 | 93 | CUGU...UCAA |
| Ef02 | URS0000AB155C_333849 | 103 | AAAC...GCUG |
| Kp01 | URS00004DF65B_1379689 | 220 | CCGU...AAAA |
| Kp04 | URS0000379E31_1379689 | 125 | UUCA...GCUG |
| Kp10 | URS00001DDDF7_1379689 | 102 | AGCG…UUGC |
| Kp11 | URS0000110F82_1379689 | 98 | UAUU…AGGG |
| Ms01^a^ | URS0000BF8745_1413510 | 109 | UUAA…UUUU |
| Ms02^a^ | URS0000BEF386_1413510 | 109 | AAUG…AGCU |
| Pa01 | URS0000AB3C94_208964 | 137 | GGGU…GCCA |
| Sa02 | URS00005EF701_1280 | 101 | CGCA…AUAU |

^a^Ms01 and Ms02 were misannotated as riboswitches from *Staphylococcus aureus*.

**Table S4: TPP levels in cell-free extracts of *Escherichia coli* MG1655 change upon addition of thiamine or pyrithiamine**

| **Cell-free extracts** | TPP [µg/L] | OD_600_ | TPP [µg/L] per OD_600_ |
| --- | --- | --- | --- |
| MG1655 grown without addition of thiamine or pyrithiamine | 468 | 5,12 | 91 |
| MG1655 grown in the presence of 10 µM thiamine | 4228 | 5,22 | 810 |
| MG1655 grown in the presence of 10 µM pyrithiamine | 418 | 5,19 | 81 |

**Figure S1: Map of the reporter plasmid pDluc.** In pDluc the (predicted) ribosomal binding sites of the TPP riboswitches to be tested replaced the ribosomal binding site (RBS) of the firefly luciferase reporter gene (*lucF*) whose start codon is shown. The origin of replication (ColE1 ori), the ampicillin resistance gene *ampR* and the corresponding promoter (prom AmpR), the *Escherichia coli* *rrnB* T1 transcriptional terminator (*rrnB* T1), the constitutive OXB15 promoter (OXB15) driving expression of the *Renilla* luciferase gene *lucR* and the relevant restriction endonuclease sites are shown as well. The *E. coli* *thiC* promoter region P_Ec_ was inserted upstream of the *lucF* gene between the *Pvu*II and *Nco*I restriction sites. Riboswitches tested in combination with the P_Ec_ promoter were inserted between the *Nco*I and *Bam*HI sites. Complete promoter-riboswitch regions and promoter control regions were inserted between the *Pvu*II and *Bam*HI sites. Notably, if the putative riboswitch reduced gene expression upon ligand treatment employing pDluc, control may have occurred at both the transcriptional and translational levels (see also Fig. S2 and Fig. S3 below).

**Figure S2: Plasmid map of pDlucTC.** Elements are the same as in Fig. S1. The *E. coli* *thiC* promoter region P_Ec_ was inserted upstream of the *lucF* gene between the *Pvu*II and *Nco*I restriction sites. Riboswitch regions tested in combination with the P_Ec_ promoter were inserted between the *Nco*I and *Bam*HI sites. Complete promoter-riboswitch regions and promoter control regions were inserted between the *Pvu*II and *Bam*HI sites. When the putative riboswitch sequence to be tested was coupled to pDlucTC and *in vivo* reduced gene expression upon ligand treatment, control occurred only at the transcription level. To distinguish between the two different mechanisms of action, the riboswitches were tested with both plasmids pDluc (Fig. S1) and pDlucTC.

**
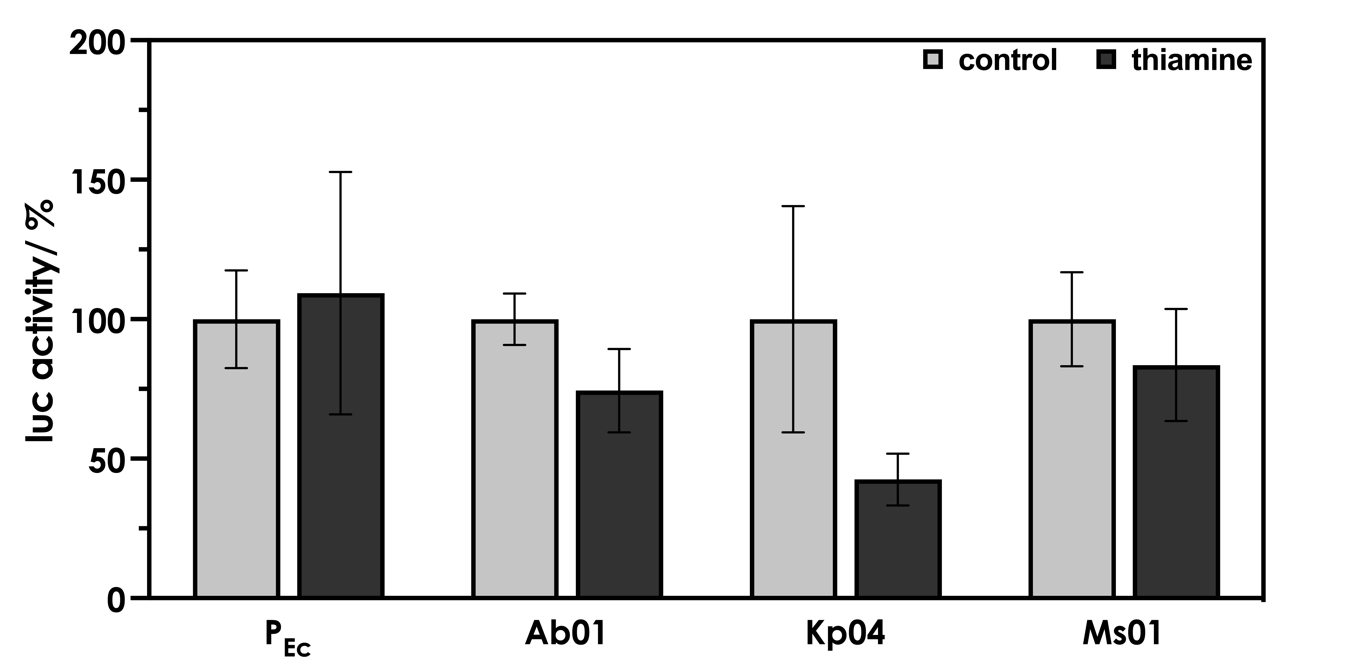
**

Figure S3: Thiamine does not negatively affect TPP riboswitches from pathogenic bacteria driven by the *E. coli* *thiC* promoter P_Ec_ when translational constructs were employed. *E. coli* strains transformed with the dual-luciferase reporter plasmid pDluc (to test for translation control) containing putative TPP riboswitches from *Acinetobacter baumannii* (*thiC*, Ab01), *Klebsiella pneumoniae* (*thiC*, Kp04) and *Mammaliicoccus sciuri* (*tenA*, Ms01) were grown in M9 minimal medium without (control) and in the presence of 10 µM thiamine. The putative TPP riboswitches were coupled to the reporter gene *lucF* and tested in combination with *E. coli* *thiC* promoter P_Ec_. The control strain contained pDluc in which expression of *lucF* was solely driven by P_Ec_ in the absence of a TPP riboswitch and thus was not affected upon addition of thiamine. Firefly luciferase (LucF) activity was normalized to constitutive *Renilla* luciferase (LucR) activity and is shown as relative activity compared to the control. Cultures were grown in triplicates in a 12-well plate. Depicted are the mean values ± standard deviations of the data obtained from triplicates. Asterisks indicate statistically significant differences (*** p ≤ 0.001).


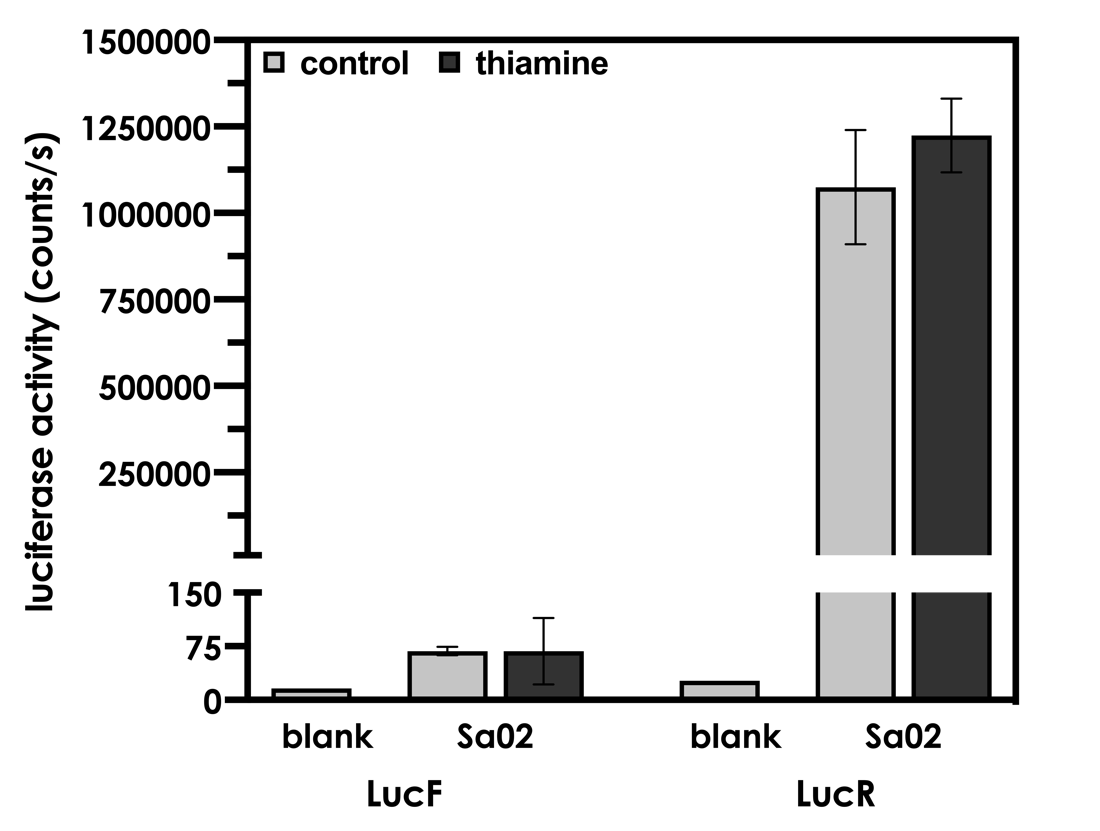


**Figure S4: The putative *Staphylococcus aureus* *thiBPQ* Sa02 riboswitch does not allow reporter gene (*lucF*) expression when tested in combination with the *E. coli* *thiC* promoter.** *E. coli* DH5α transformed with the dual-luciferase reporter plasmid pDluc containing the putative *S. aureus* *thiBPQ* Sa02 TPP riboswitch (Sa02) under control of the *E. coli* *thiC* promoter was grown in M9 in the absence (control) and presence of 10 µM thiamine. Firefly luciferase (LucF) and *Renilla* luciferase (LucR) activity was measured in counts/s. LucF activity is very low showing that Sa02 does not allow reporter gene expression when placed downstream of the *E. coli* *thiC* promoter. The gene encoding LucR is constitutively expressed from p_OXB15_ and its expression is not affected by thiamine. Cultures were grown in triplicates in a 12-well plate. Depicted are the mean values ± standard deviations of the data obtained from the triplicates. Single blank measurements performed with 1x PLB buffer are shown as well.


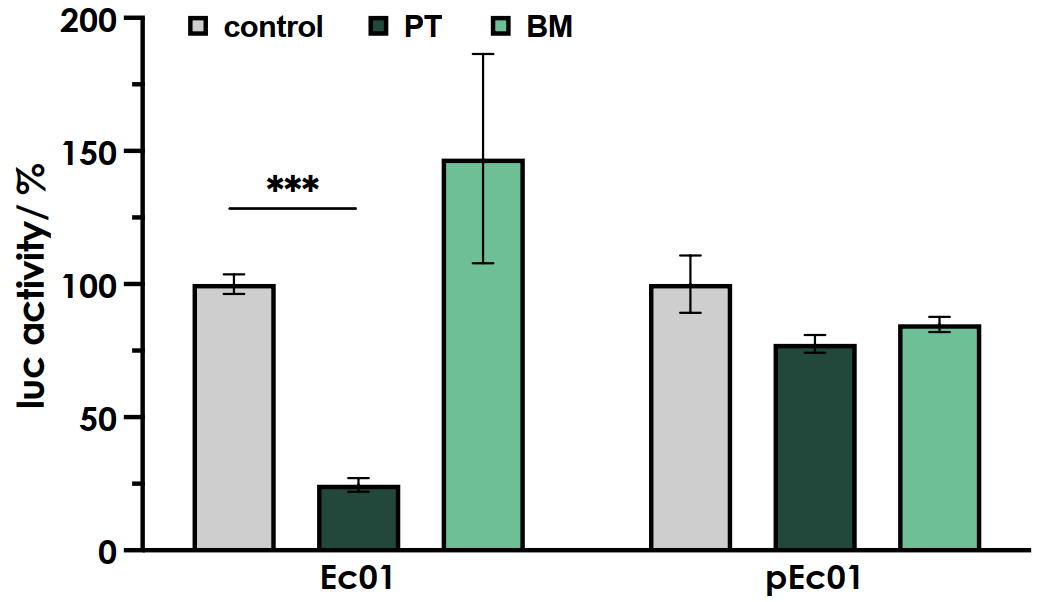


**Figure S5: Addition of pyrithiamine but not of the natural thiamine analog bacimethrin negatively affects the *Escherichia coli thiC* riboswitch (Ec01) under control of its natural promoter pEc01*.*** The TPP riboswitch Ec01 under control of the promoter pEc01 was coupled to pDluc and the corresponding *E. coli* MG1655 test strain was challenged with 10 µM pyrithiamine (PT) or 10 µM bacimethrin (BM). The control was grown in the absence of pyrithiamine or bacimethrin. The promoter control, not containing a TPP riboswitch, is shown in the right panel. Firefly luciferase (LucF) activity was normalized to constitutive *Renilla* luciferase (LucR) activity and is shown as relative activity compared to the controls. Cultures were grown in triplicates in a 12-well plate. Depicted are the mean values ± standard deviations of the data obtained from the triplicates. Asterisks indicate statistically significant differences (*** p ≤ 0.001).


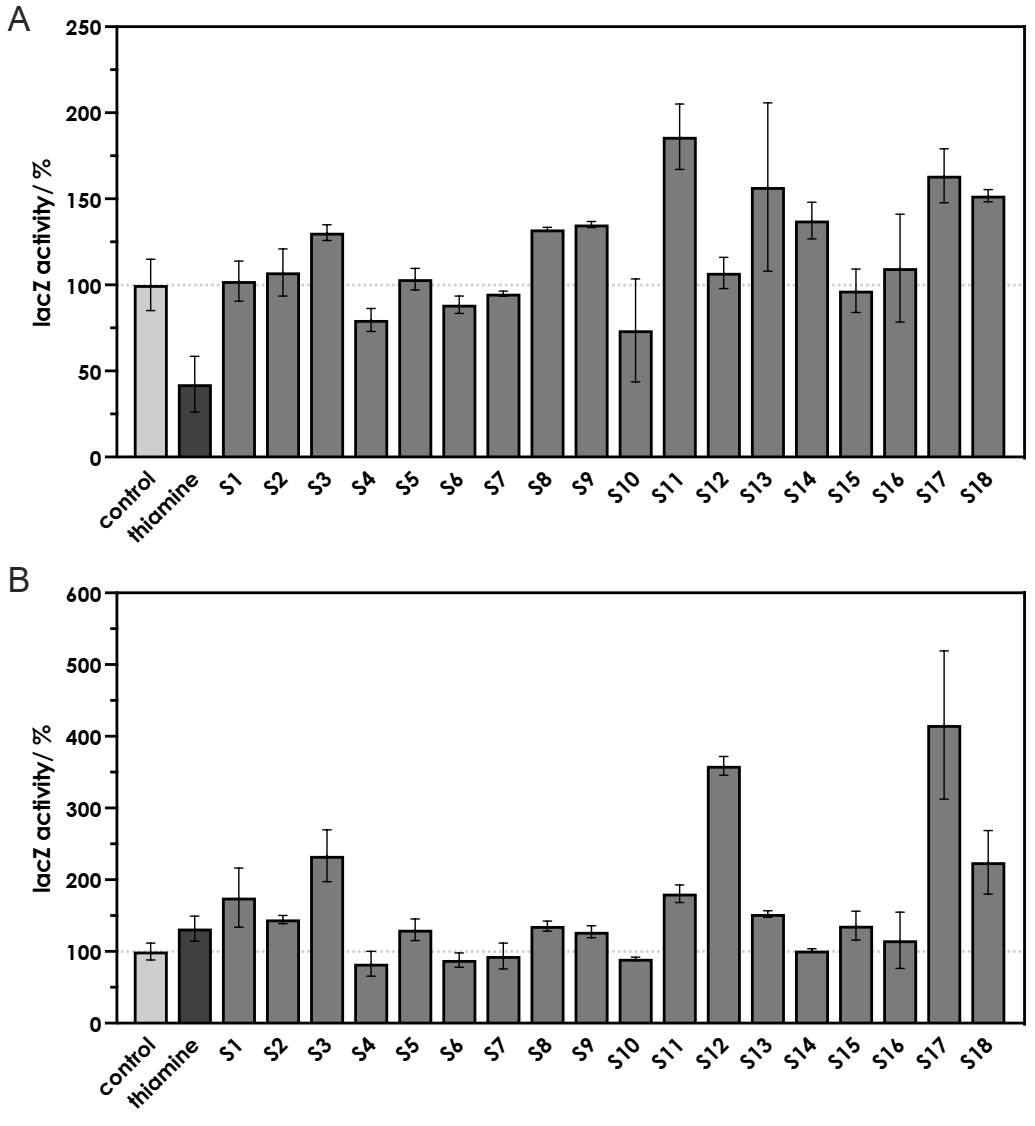


Figure S6: Possible effectors of the K. pneumoniae thiC riboswitch Kp04 **were tested in an *in vivo* assay.** E. coli DH5α strains transformed with the pHA191 reporter plasmid were grown in M9 minimal medium in the absence (control) or presence of 1 mM of thiamine or possible effectors (see Fig. S6). LacZ activity was measured using ortho*-*nitrophenyl-β-D-galactopyranoside as a substrate. In strain Kp04 the native K. pneumoniae promoter thiC is coupled to the riboswitch thiC. Reduced *lacZ* expression upon addition of thiamine indicates that thiamine turns the riboswitch off. In strain pKp04 the reporter gene is constitutively expressed from the K. pneumoniae thiC promoter as no riboswitch is present. LacZ activity is given as relative activity compared to the control. Cultures were grown in triplicates in a plate reader in a 96-well plate. Depicted are the mean values ± standard deviations of the data obtained from the triplicates.


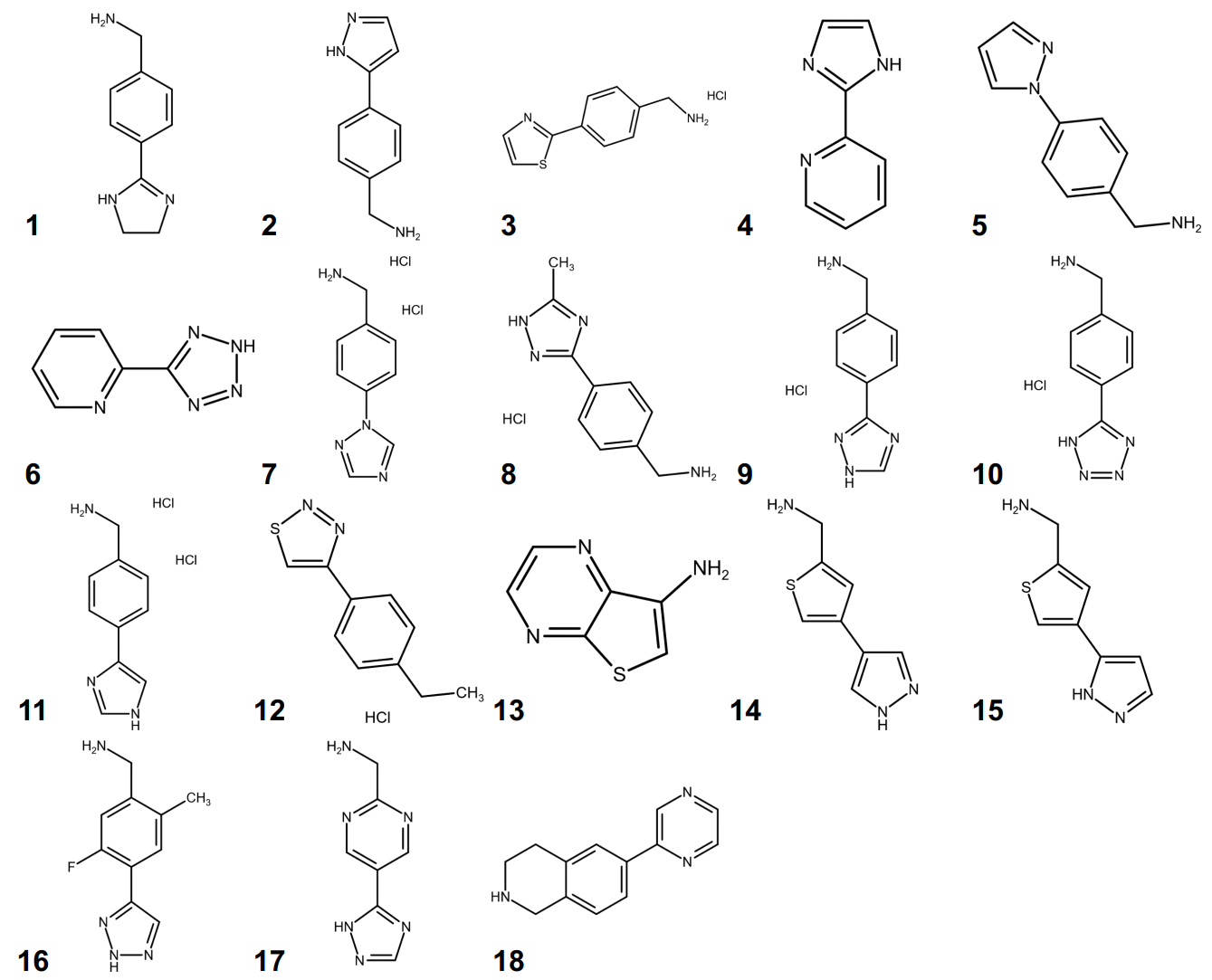


Figure S7: Possible effectors of the TPP riboswitch Kp04 of *Klebsiella pneumoniae* tested using a *lacZ* based reporter gene construct. **1**, 1-[4-(1*H*-imidazol-2-yl)phenyl] methanamine; **2**, 1-[4-(1*H*-pyrazol-5-yl)phenyl] methanamine; **3**, 1-[4-(1,3-thiazol-2-yl)phenyl] methanamine hydrochloride; **4**, 2-(1*H*-imidazol-2-yl) pyridine; **5**, 1-[4-(1*H*-pyrazol-1-yl)phenyl] methanamine; **6**, 4-(2*H*-tetrazol-5-yl) pyridine; **7**, 1-[4-(1*H*-1,2,4-triazol-1-yl)phenyl] methanamine dihydrochloride; **8,** 1-[4-(5-methyl-1*H*-1,2,4-triazol-3-yl)phenyl] methanamine hydrochloride; **9**, 4-(1*H*-1,2,4-triazol-3-yl)phenyl] methanamine hydrochloride; **10**, 4-(2*H*-tetrazol-5-yl)-benzenemethanamine hydrochloride; **11**, 1-[4-(1*H*-imidazol-4-yl)phenyl] methanamine dihydrochloride; **12**, 4-(1,2,3-thiadizol-4-yl) benzylamine hydrochloride; **13**, thieno [2,3-b]pyrazin-7-amine; **14**, 1-[4-(1*H*-Pyrazol-4-yl)-2-thienyl] methanamine; **15**, 1-[4-(1*H*-pyrazol-3-yl)-2-thienyl] methanamine; **16**, 1-[5-fluoro-2-methyl-4-(1*H*-1,2,3-triazol-5-yl)phenyl] methanamine; **17**, 1-[5-(4*H*-1,2,4-triazol-3-yl)-2-pyrimidinyl] methanamine; **18**, 6-(pyrazin-2-yl)-1,2,3,4-tetrahydroisoquinoline.


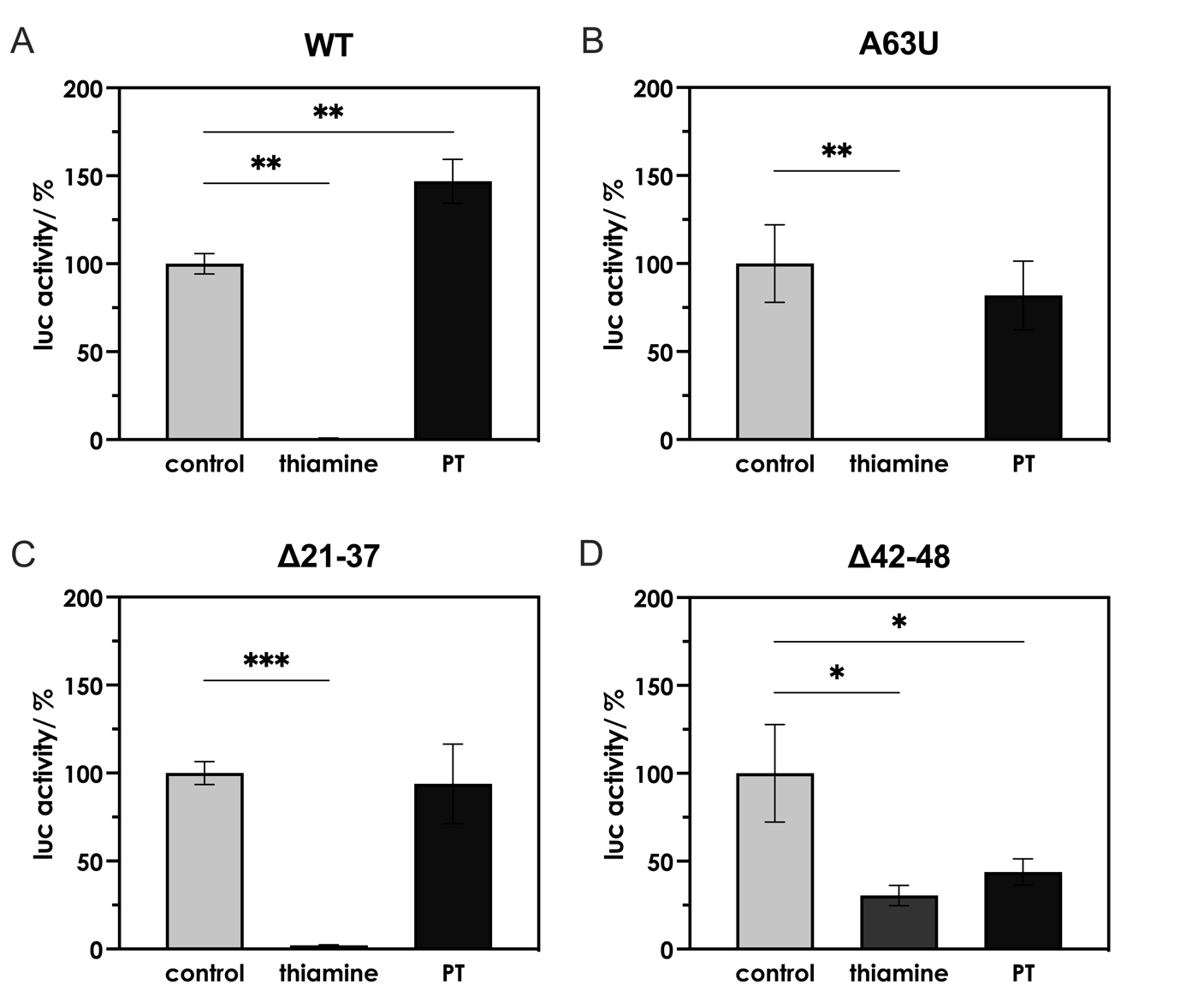


Figure S8: Effects of thiamine and pyrithiamine on the *K. pneumoniae thiC* riboswitch (A) and its mutants Kp04^A63U^ (B), Kp04^Δ21-37^ (C) and Kp04^Δ42-48^ (D). Test plasmids based on pDluc containing Kp04 wild-type and variants thereof coupled to the *K. pneumoniae thiC* promoter were used to transform *E. coli*. The resulting test strains were grown in M9 in the absence or presence of 10 μM thiamine or pyrithiamine (PT). LucF activity was normalized to LucR activity and is given as relative activity compared to the control. Cultures were grown in triplicates in a 12-well plate. Depicted are the mean values ± standard deviations of the data obtained from the triplicates. Asterisks indicate statistically significant differences (* p ≤ 0.05, ** p ≤ 0.01, *** p ≤ 0.001).


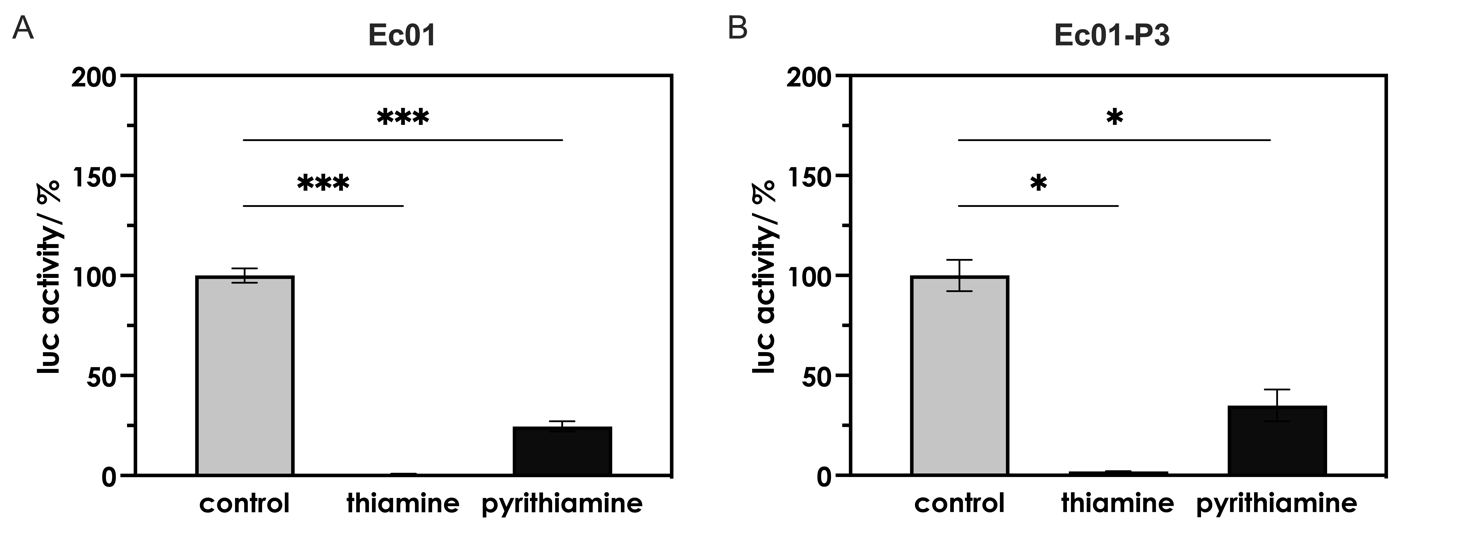


Figure S9: Response of the *E. coli* *thiC* riboswitch Ec01 to pyrithiamine (A) does not change upon insertion of the P3 stem of the *K. pneumoniae thiC* riboswitch to the *E. coli* *thiC* riboswitch (Ec01-P3, B). The reporter plasmid pDluc::Ec01-P3 was created by inserting the P3 stem of the *K. pneumoniae thiC* riboswitch Kp04 (ttc cat gcg cag gct aac gtg gaa) between nucleotides 18 and 19 of the *E. coli* *thiC* riboswitch Ec01. *E. coli* MG1655 containing the test plasmid pDluc::Ec01 (A) or pDluc::Ec01-P3 (B) was grown in M9 in the absence or presence of 10 μM thiamine or pyrithiamine (PT). When compared to the results obtained with *E. coli* Ec01(A) no difference with regard to LucF activity was found for Ec01-P3 (B) showing that addition of P3 does not change the susceptibility with regard to pyrtithiamine. LucF activity was normalized to LucR activity and is given as relative activity compared to the control. Cultures were grown in triplicates in a 12-well plate. Depicted are the mean values ± standard deviations of the data obtained from the triplicates. Asterisks indicate statistically significant differences (* p ≤ 0.05).


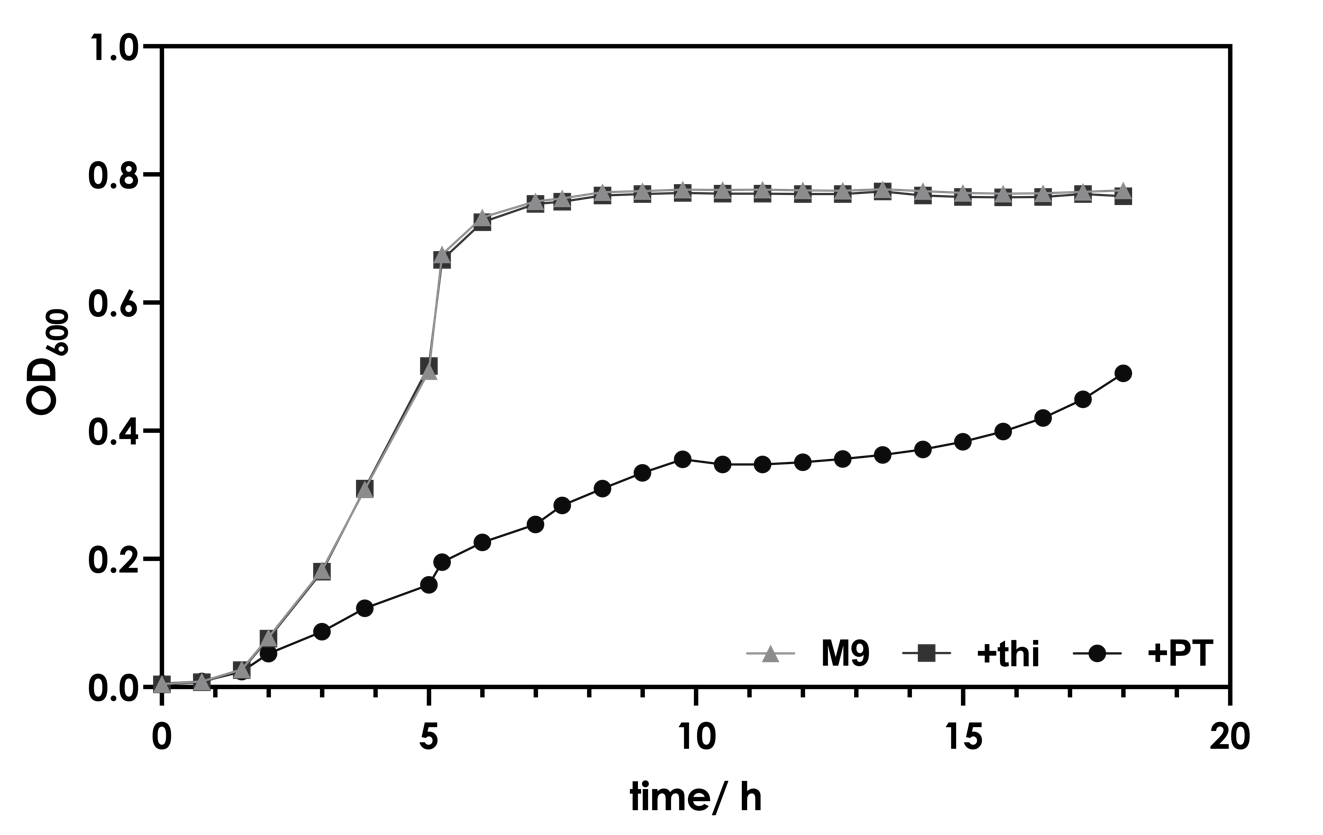


Figure S10: Effect of thiamine or pyrithiamine on growth of *Klebsiella pneumoniae* in M9. Cultures of *K. pneumoniae* were grown in M9 in the absence (M9, light-gray triangles) or presence of either 300 µM thiamine (thi, gray squares) or pyrithiamine (PT, black circles). The cultures (1 mL) were grown in triplicates in 24-well plates and growth was monitored by measuring the optical density of the suspensions at λ=600 nm. Depicted are the mean values of the data obtained from the triplicates. The standard deviations were too small and are therefore not recognizable as error bars in the diagram.


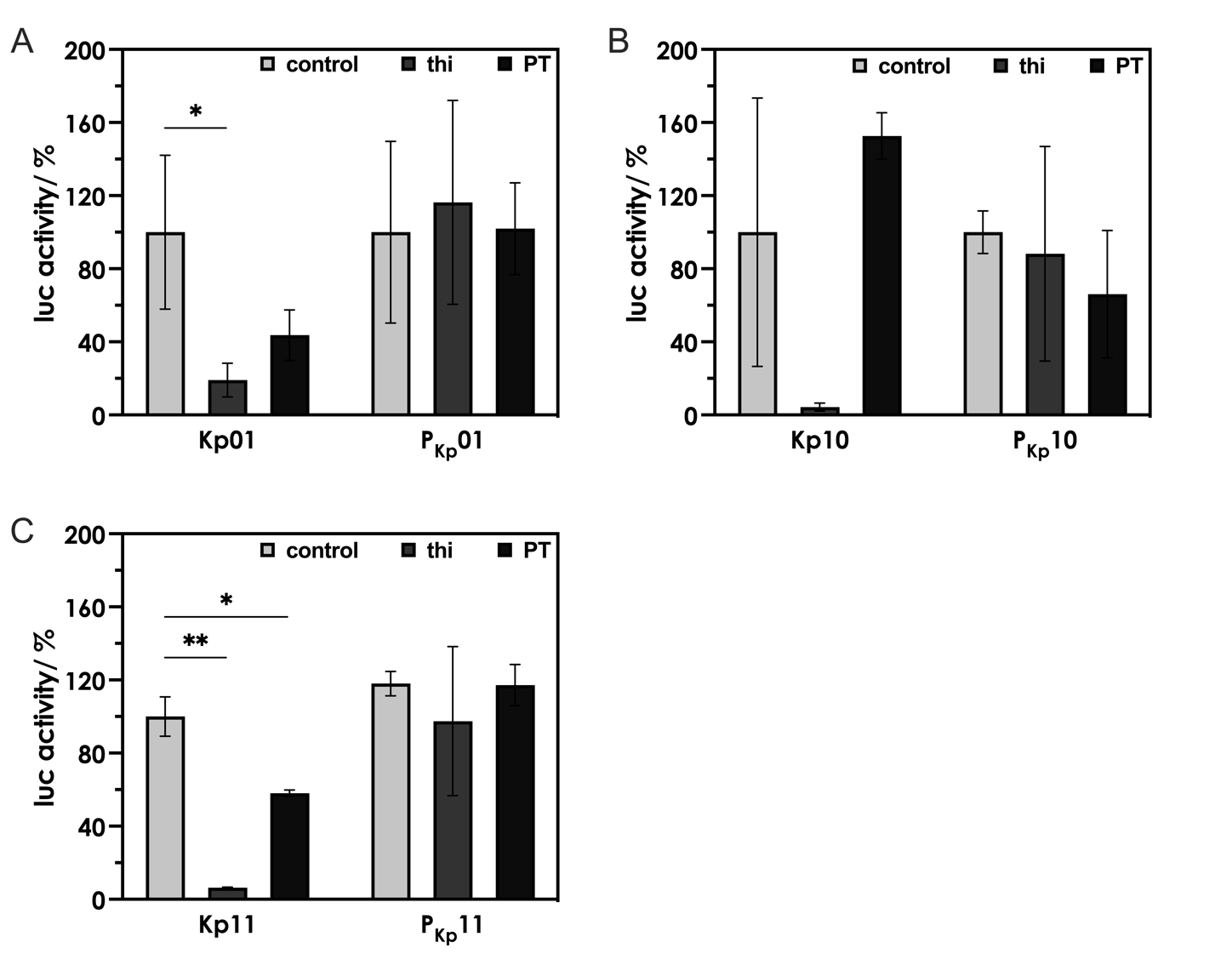


Figure S11: Effect of thiamine and pyrithiamine on the activity of the *K. pneumoniae* TPP riboswitches *thiBPQ*, *tenA* and *thiM* (C). Test plasmids based on pDluc containing the *K. pneumoniae* TPP riboswitches *thiBPQ* (Kp01) (A), *tenA* (Kp10) (B) and *thiM* (Kp11) were coupled to the *K. pneumoniae thiC* promoter and used to transform *E. coli*. The resulting test strains were grown in M9 in the absence or presence of 10 μM thiamine or pyrithiamine (PT). LucF activity was normalized to LucR activity and is given as relative activity compared to the control. Cultures were grown in triplicates in a 12-well plate. Depicted are the mean values ± standard deviations of the data obtained from the triplicates. Asterisks indicate statistically significant differences (* p ≤ 0.05, ** p ≤ 0.01).


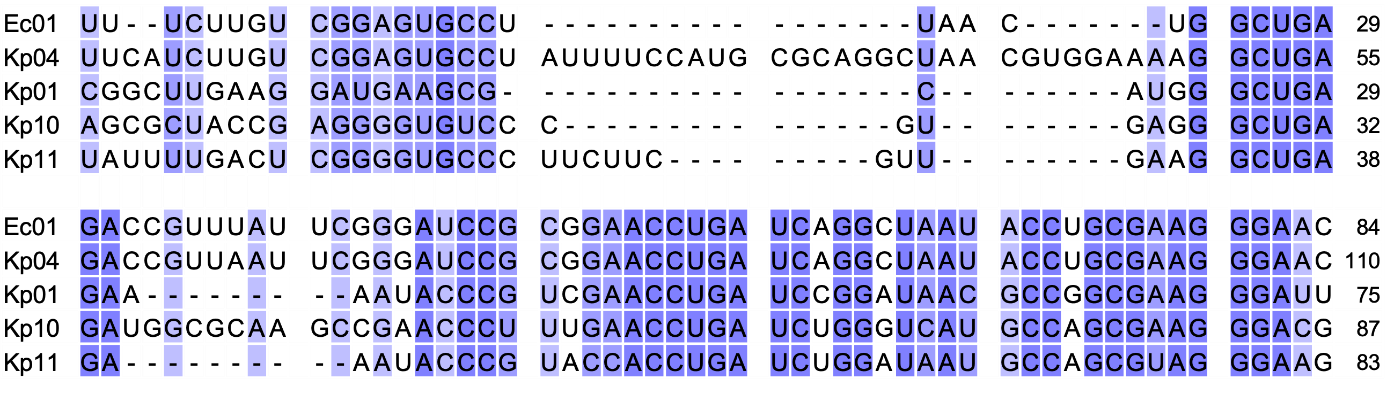


Figure S12: Alignment of aptamer sequences of the *E. coli* *thiC* (Ec01) and *K. pneumoniae* *thiC* (Kp04), *thiBPQ* (Kp01), *tenA* (Kp10) and *thiM* (Kp11) riboswitches. Sequences were aligned using the R-Coffee web server (1). Conserved nucleotides are highlighted in blue; the deeper the blue, the higher the degree of conservation.


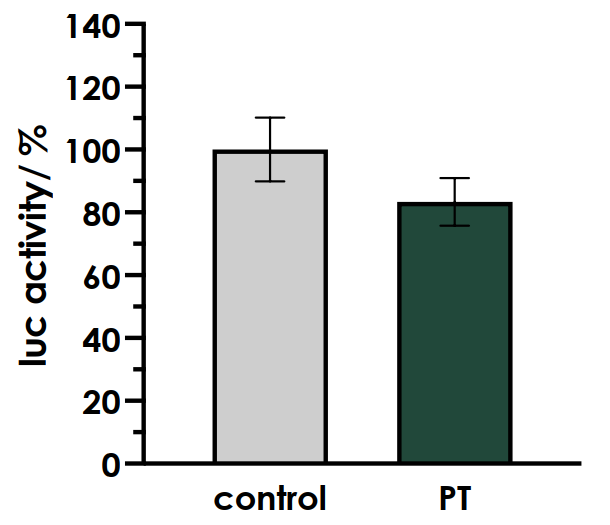


Figure S13: Pyrithiamine does not affect the *Klebsiella pneumoniae* riboswitch Kp04 in combination with the *Escherichia* *coli* *thiC* promoter. The TPP riboswitch Kp04 (wild-type) under control of the *E.* *coli* *thiC* promoter was coupled to pDluc and the corresponding *E. coli* MG1655 test strain was challenged with 10 µM pyrithiamine (PT). The control was grown in the absence of pyrithiamine. Firefly luciferase (LucF) activity was normalized to constitutive *Renilla* luciferase (LucR) activity and is shown as relative activity compared to the controls. Cultures were grown in triplicates in a 12-well plate. Depicted are the mean values ± standard deviations of the data obtained from the triplicates.

References

1. Moretti S, Wilm A, Higgins DG, Xenarios I, Notredame C. 2008. R-Coffee: a web server for accurately aligning noncoding RNA sequences. Nucleic Acids Res 36:W10-3. doi:10.1093/nar/gkn278.
